# Bioengineering Human Neurological Constructs Using Decellularized Meningeal Scaffolds for Application in Spinal Cord Injury

**DOI:** 10.3389/fbioe.2018.00150

**Published:** 2018-11-01

**Authors:** Sandeep Kumar Vishwakarma, Avinash Bardia, Chandrakala Lakkireddy, Syed Ameer Basha Paspala, Aleem Ahmed Khan

**Affiliations:** ^1^Central Laboratory for Stem Cell Research and Translational Medicine, CLRD, Deccan College of Medical Sciences, Kanchanbagh, Hyderabad, India; ^2^Dr. Habeebullah Life Sciences, Hyderabad, India

**Keywords:** bioengineered neuronal construct, decellularized meningeal scaffolds, spinal cord injury, hNPCs, nervous tissue regeneration

## Abstract

Spinal cord injury (SCI) is one of the most devastating conditions echoes with inflammation, enhanced fibrosis and larger axonal gaps due to destruction of neurological cells which has caused continuous increasing mortality rate of SCI patients due to absence of suitable treatment modalities. The restoration of structural and functional aspect of damaged neurological tissues at the lesion site in spinal cord has been challenging. Recent developments have showed tremendous potential of neural stem cell-based strategies to form a neuronal relay circuit across the injury gap which facilitates some levels of improvement in SCI condition. However, to provide better therapeutic responses, critical mass of grafted cells must survive for long-term and differentiate into neuronal cells with well-developed axonal networks. Hence, development of tissue specific biological neuronal constructs is highly desirable to provide mechanical and biological support for long-term survival and function of neurological cells within natural biological niche. In this study, we report development of a tissue specific neuronal constructs by culturing human neural precursor cells on decellularized meningeal scaffolds to provide suitable biological neuronal construct which can be used to support mechanical, structural and functional aspect of damaged spinal cord tissues. This particular tissue specific biological construct is immunologically tolerable and provides precisely orchestral three-dimensional platform to choreograph the long-distance axonal guidance and more organized neuronal cell growth. It passes sufficient mechanical and biological properties enriched with several crucial neurotrophins required for long-term survival and function of neurological cells which is required to form proper axonal bridge to regenerate the damaged axonal connectomes at lesion-site in SCI.

## Introduction

Spinal cord injury (SCI) has been one of the most devastating conditions with increasing mortality rates due to lack of suitable treatment modalities. The major functional deficits following SCI represent pathologies of axonal damage, demyelination and loss of neuronal cells. In addition to these initial mechanical insults, secondary pathological mechanisms such as ischemia, free radical formation, development of an inhibitory extracellular matrix (ECM) around the site of injury, lack of growth promoting factors such as neurotrophins contribute to failure of neuronal tissue regeneration after SCI (He and Koprivica, 2004; Schwab, 2004; Fawcett, 2006; Fitch and Silver, 2008; Lu and Tuszynski, 2008). Despite advances in several strategies, structural and functional restoration of damaged axonal networks at lesion site in SCI remains elusive (Blesch and Tuszynski, 2009). Since many years, enormous efforts have been made to overcome these challenges and find suitable treatment strategy for structural and functional support to bridge the gap in SCI.

The recent advances in stem cell-based therapies have opened a new avenue to explore better possibility for developing effective treatment strategies in SCI (Vismara et al., 2017; Liu et al., 2018). Transplantation of human neural precursor cells (hNPCs) has demonstrated high therapeutic potential for reconstruction of damaged axonal networks at the lesion site in spinal cord. The studies have demonstrated that grafting of NPCs at injury site forms a neuronal relay circuit across the injury gap and facilitates improvement in SCI (Jakeman and Reier, 1991; Reier et al., 1992; Coumans et al., 2001; Cummings et al., 2005; Salazar et al., 2010; Bonner et al., 2011). Furthermore, transplanted NPCs may also reduce secondary complications (Cummings et al., 2005) and have potential to re-myelinate spared, demyelinated axonal networks (Keirstead et al., 2005; van den Brand et al., 2012; Tohyama et al., 2017; Rosenzweig et al., 2018). However, to provide better therapeutic responses, critical mass of grafted cells must survive for long-term and differentiate into neurons, support the growth of injured host axons into the graft, accept synapses from host derived neurons, extend neurons into the injured spinal cord and provide mechanical support (Courtine et al., 2008). Therefore, transplantation of NPCs alone could not provide required mechanical strength and hospitable microenvironment for long-term support of axonal growth and function (Badner et al., 2017). Hence, developing suitable immunocompatible biological scaffolds with adequate mechanical and biological properties are desired to support the long-term growth and differentiation of human NPCs to improve the structural and functional benefits at injury site. However, finding such biomimetic neuronal constructs for proper structural and functional support of the damaged spinal cord has always been challenging. Recently several kinds of fabricated three-dimensional (3D) scaffolds such as fibrin scaffolds, polymer-based scaffolds and nanostructured scaffolds have been proposed in neural tissue reconstruction (Bozkurt et al., 2010; Du et al., 2011; Han and Cheung, 2011; Gao et al., 2013; Assunção-Silva et al., 2015). However, these approaches do not mimic with the natural nervous tissues architecture and cellular expansion on such scaffolds. Majority of these scaffolds may induce the additional fibrotic or immunological response at the injury site which renders their *in situ* applicability as complete biocompatible neuronal construct to reconnect the damaged neuronal axons. Hence there is need to develop more authentic biologically compatible natural human scaffolds for proper alignment and growth of interconnected functional neuronal cells which could mimic with the natural developmental mechanisms similar to the human system.

To address these needs, here we report development of biologically compatible human neuronal constructs using decellularized meningeal scaffolds (DMS) as a 3D-platform for differentiating hNPCs. The DMS harboring differentiated human neuronal cells has been termed as meningeal neuronal construct (MNC). This MNC allows accurate replication of the natural developmental processes, spatial arrangement and functionally interconnected axonal networks. This approach offers suitable 3D-microarchitecture and more hospitable microenvironment enriched with several crucial neurotrophins required for long-term cell survival and function. This particular strategy may overcome on certain limitations of earlier developed synthetic biomaterials in terms of mechanical properties, natural 3D-extracellular brain matrix, growth factors, and supplements resulting in favorable biological compatibility to restore the damaged neuronal networks in SCI. This strategy imitates a precisely orchestral platform to support tissue specific neuronal construct for organized neuronal cell growth which is required to provide sufficient mechanical and biological support by providing proper axonal bridge to complete the damaged neuroconnectomes at lesion-site in SCI.

## Results

**Figure 1:**
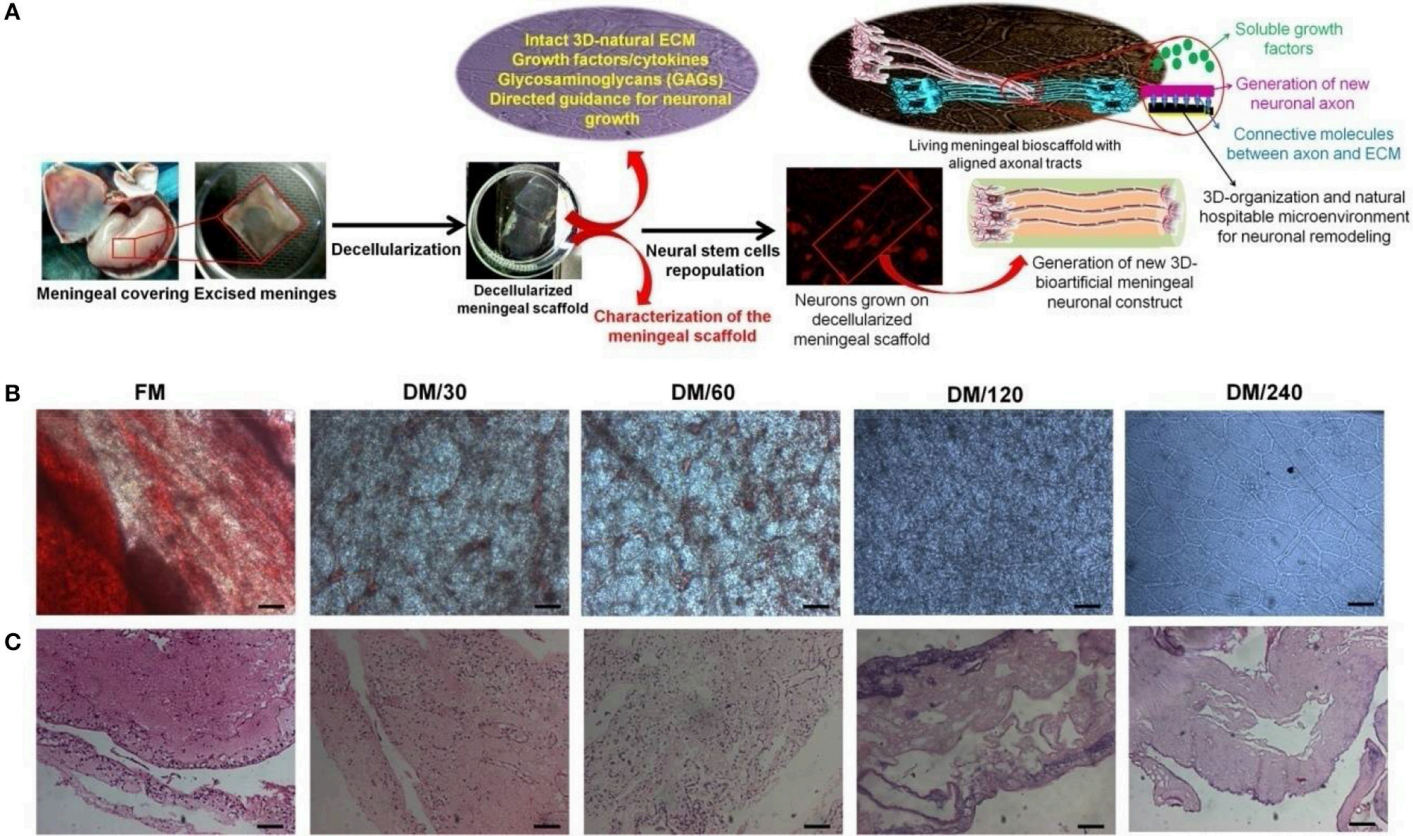
**(A)** Schematic representation showing the strategy for development of bioengineered humanized neuronal constructs using decellularization and repopulation strategy. This meninegal neuronal construct (MNC) is comprised of human neuronal cells having well developed axonal tracts on decellularized meningeal scaffolds (DMS). **(B)** Microscopic analysis showing the changes in the phenotype during decellularization process of human brain meninges. **(C)** H&E stained micro-sections showing elimination of nuclear contents and preservation of ECM and natural architecture during decellularization of native/fresh meninges (FM) at different time points. (Scale bar: 40 μm; Resolution: 10X). FM, fresh/native meninges; DM/30, decellularized meninges after 30 min; DM/60, decellularized meninges after 60 min (1 h); DM/120, decellularized meninges after 120 min (2 h); DM/240, decellularized meninges after 240 min (4 h).

The development of 3D-tissue specific niche has been performed using decellularization and repopulation strategy. The resulting DMS has been utilized for generating MNC by repopulating differentiated hNPCs (Figure 1A). This representation was drawn to provide realistic overview for providing bio-mimetic 3D-neurological construct to support structural and functional cues involved in neurogenic regeneration at lesion-site. DMS described herein provides native 3D-ECM, essential growth factors for neural cells engraftment at defined locations, tissue specific spatial organization, long-term survival, lineage differentiation, and directed axonal growth which are essential to develop extended neuronal networks for providing more appropriate biological construct for SCI regeneration.

### Characterization of decellularized meninges

#### Optical and microscopic analysis of DMS

DMS were generated using the process of detergent-based decellularization and further tested for the retention of cell free, intact tissue specific ECM, and natural 3D-architecture of the human meningeal tissues. The procedure followed in this study generates completely DMS within 240 min. Optical observation of meningeal tissues during decellularization process showed increasing translucent appearance due to dissolution of cells with increasing the time (Figure 1B). Microscopic analysis demonstrated the clearance of cellular materials with increasing the incubation time of decellularization during switch over of gradients of decellularization solutions. Figure 1B clearly shows that fresh meninges (FM) have intact vascular web with red blood cells (RBCs) and other meningeal cells on the ECM which gives it very blunt appearance under light microscope. However, during decellularization process the amount of RBCs and other type of cells gets reduced with increasing the time due to enzymatic lysis and mechanical pressure. After 240 min, DMS showed clear and intact vascular networks with completely translucent appearance.

#### Immunohistolochemical analysis of DMS

**Figure 2:**
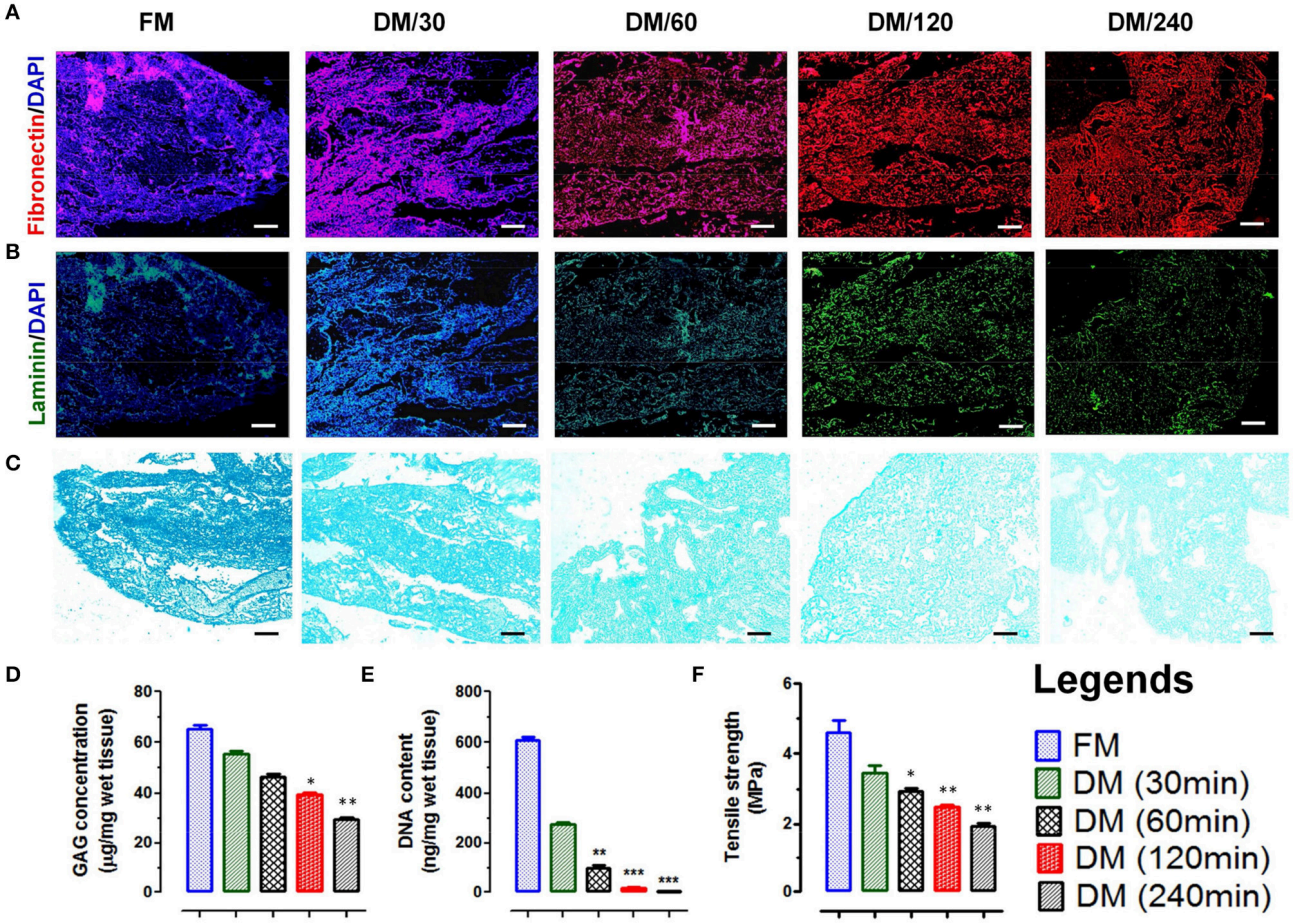
Immunofluorescence staining of native and decellularized meningeal tissue micro-sections showing presence and absence of nuclear staining (DAPI, blue in color) with retention of natural architecture of ECM components such as **(A)** fibronectin (red) and **(B)** laminin (green). (Scale bar: 40 μm; Resolution: 10X). **(C)** Alcian blue staining of FM and DM also showed positive staining for GAG. The staining intensity was reduced with increasing the time of decellularization process. However, the staining pattern was similar throughout the material (Scale bar: 40 μm; Resolution: 10X). **(D)** The preservation of GAGs in DMS showed continuous decrease with increasing time of decellularization process and reduced up to 50% after 240 min (DM/240). **(E)** Quantification of residual DNA content in meninges showed continuous decrease in DNA quantity with increasing the time of decellularization and was almost negligible after 240 min (DM/240). **(F)** The tensile strength of decellularized meninges reduced up to 50% after complete decellularization (DM/240). ^*^p < 0.01, ^**^p < 0.001, ^***^p < 0.0001.

Imunohistolochemical analysis of DMS before and after decellularization at different time points was performed using Hematoxylene and Eosin (H&E) and specific antibodies staining for fibronectin and laminin. The histological evaluation of H&E stained sections of DMS showed gradual decrease of cells and its components with increasing the decellularization time and were completely absent after 240 min (Figure 1C). H&E staining also revealed preservation of tissue specific ECM and vascular architecture within completely DMS similar to the fresh meningeal tissues. Immunohistochemical analysis of preserved ECM components within the DMS showed positive staining for the expression of fibronectin (Figure 2A and Supplementary Figure 2.1) and laminin (Figure 2B and Supplementary Figure 2.2). This observation revealed that the decellularization process followed herein, fully maintains the tissue specific ECM proteins similar to the FM. Importantly, the ECM distribution within the DMS confirmed the packed preservation of architectural pattern similar to native ones.

#### Residual glycosaminoglycans content in DMS

The existence of native glycosaminoglycans (GAGs) within DMS was determined using Alcian blue staining which showed the preservation of GAG content throughout the decellularization process (Figure 2C). The quantitative analysis of GAGs present in ECM of DMS revealed almost 50% existence of GAGs after complete decellularization process (Figure 2D) which is essential for cell to ECM interaction for enhanced cell adhesion and survival.

#### Retention of nuclear contents within DMS

The absence of nucleic acids in DMS was analyzed by estimating DNA content using nucleic acid quantification. The absorbance of residual nucleic acid content in tissue lysate measured at 260 and 280 nm showed continuous decrease in DNA content with increasing the decellularization time and was almost negligible after 240 min (Figure 2E). The DNA quantity was significantly lower in DMS after 240 min as compared with the FM (^***^p < 0.0001).

#### Mechanical properties of DMS

Mechanical integrity of DMS was determined by tensile strength analysis using tensile test. The tensile strength of native meninges (FM) was higher (4.8 ± 0.75 MPa) than the DMS which was continuously decreased with increasing the decellularization time and retained up to 50% after complete decellularization process (240 min) which was measured ~2.8 ± 0.51 MPa (Figure 2F). The retention of 50% mechanical strength of DMS provides more flexibility for cellular adherence and engraftment efficiency within the DMS.

#### Ultra-structure analysis of DMS

**Figure 3:**
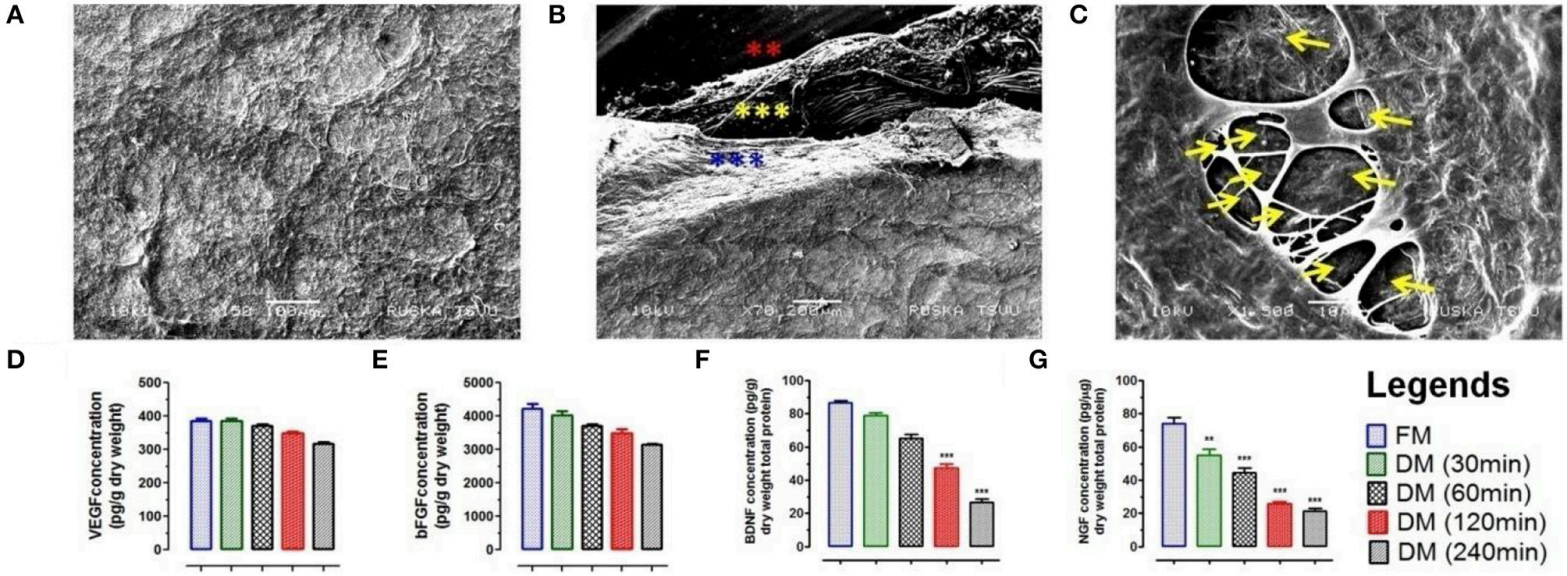
**(A)** Ultra-structure analysis using SEM analysis of DMS (DM/240) showed intact architecture of tissue specific ECM and 3D-organization to provide suitable microenvironment for neuronal cells survival and expansion. **(B)** SEM analysis revealed fibrous micro-structures of meningeal tissue in native form. The 3D network of connective tissue fibers were arranged in specific manner in native form through the network of ECM proteins. All the three different layers of meninges are depicted by star marks in different colors. **(C)** Cross section of DMS (DM/240) showing presence of vascular network within the scaffold (depicted by yellow color arrows). Quantification of retained cytokines **(D)** VEGF and **(E)** bFGF in DMS showed significant higher amount of retention even after 240 min of decellularization which was almost similar to the native meningeal tissue (FM) (*p* > 0.05). However, significant decrease was observed for **(F)** BDNF and **(G)** NGF with increasing time of complete decellularization (^**^p < 0.001, ^***^p < 0.0001).

Scanning electron microscopy (SEM) was performed to evaluate the impact of meningeal decellularization on microstructures such as collagen fibers, fibronectin, and laminin (seen around the vasculature) and 3D-architecture of ECM in completely DMS. Ultra-structural analysis of DMS (after 240 min) surface showed retention of highly intact micro-structures of ECM specific proteins and tissue architecture (Figure 3A). The key multilayered architectural structures of ECM within the DMS were clearly recognized with their peculiar features and arrangements in highly specialized manner (Figure 3B). All three meningeal layers can be easily distinguished in from of dural layer (blue asterisks), arachnoid (yellow asterisks), and pia matter (red asterisks). The 3D network of connective tissue fibers were arranged in specific manner in native form through the network of ECM proteins. The cross-section of DMS in SEM analysis showed preservation of highly intact larger and smaller vascular tracts (Figure 3C). The dense organization of structural proteins and fibers gives very blunt appearance, however DMS were found to have porous structures as observed in cross section (Figure 3C). Overall, the SEM analysis confirmed the preservation of 3D meningeal microanatomy and ultra-structures after complete decellularization process.

#### Preservation of active biological molecules within DMS

The retention of natural growth factors and cytokines in DMS was quantified in extracted proteins by enzyme-linked immunosorbent assay (ELISA). This analysis showed preservation of significantly high amount of vascular endothelial growth factor (VEGF) (Figure 3D), basic fibroblast growth factor (bFGF) (Figure 3E), brain-derived neurotrophic factor (BDNF) (Figure 3F) and nerve growth factor (NGF) (Figure 3G) within DMS. The levels of VEGF and bFGF within DMS after 240 min were almost similar with respect to FM (*p* > 0.05). However, the levels of BDNF and NGF were significantly reduced after complete decellularization (240 min) of meningeal scaffolds with respect to FM (^***^p < 0.0001). The significant reduction in BDNF and NGF might be due to the loss of neurological cells and their components during the decellularization process. The retention of VEGF within DMS can be described in term of preservation of highly vascularized structures within the scaffolds while bFGF is an ECM-bound protein which is essential for ECM to cellular interaction.

### DMS provides 3D-orchestral platform for human neuronal cells to choreograph the synchronized axonal growth and signaling

**Figure 4:**
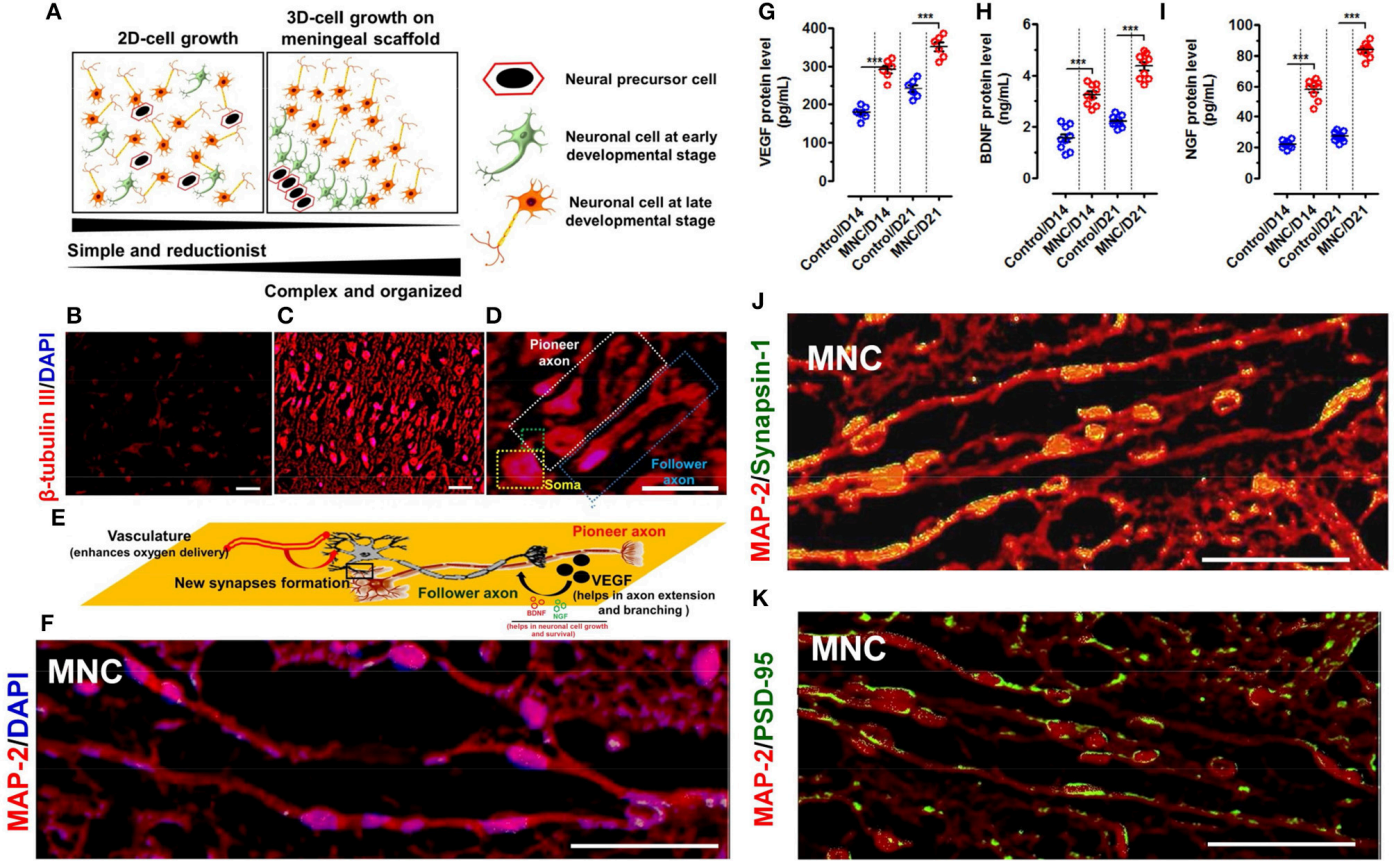
**(A)** Schematic representation showing the spatial arrangement of neuronal cells in 2D and 3D-culture system. The neurological cells in 2D-culture is simple, reductionist and do not have specific natural arrangement. Whereas, neurological cells grown on DMS tend to arrange in 3D-architecture in more organized manner mimicking with the natural system. β-tubulin III immunofluorescence staining of neuronal cells cultured in **(B)** 2D system (i.e., on fibronectin coated coverslips) are distributed in simplified manner whereas **(C)** on DMS in 3D microenvironment they are well organized as per the perception with well-developed axons (Magnification: 20X, Scale bar: 50 μm). **(D)** High resolution fluorescence images of neurons grown on DMS showing structural cues directing the long axonal outgrowth along the pioneer axonal tract and formation of new synapses due to spatial configuration of neurons and scaffold in support of growth factors and cell adhesion molecules. The pioneer and follower axons can be easily seen on DMS (Magnification: 40X, Scale bar: 50 μm) **(E)** Schematic representation of the structural and functional cues directing the formation of follower new long stretched axonal tracts with respect to the existing pioneer axons. **(F)** Immunofluorescence image of neuronal cells grown on DMS (i.e., MNC) showing MAP-2 protein staining (red) in cultured cells along with DAPI (blue) (Scale Bar: 100 μm). Quantification of secreted cytokines **(G)** VEGF **(H)** BDNF and **(I)** NGF in culture supernatants of neurological cells grown on DMS (i.e., MNC) showing significantly higher level as compared to control condition at day 14 and day 21 (^***^p < 0.0001) **(J)** Immunofluorescence analysis showing punctate distribution of Synapsin 1 protein (green) and MAP-2 protein (red) in cultured human neuronal cells on DMS at day 21 (Scale Bar: 100 μm) **(K)** Immunofluorescence analysis showing Post Synaptic Density 95 kDa (PSD-95 or SAP90) staining (green) along with MAP protein (red) in neuronal cells cultured on DMS at day 21 (Scale Bar: 100 μm).

Small fragments of DMS (2 × 2 cm^2^) were prepared by scalpel cleavage of the whole DMS and seeded with hNPCs enriched with prominin-1 (Supplementary Figures 4.1–4.3) at the density of 1.5 × 10^5^ per cm^2^ area to develop human MNC. Cell viability on DMS was found to be 95.42 ± 4.31 percentage after 24 h of seeding which was slightly higher than the control condition (86.11 ± 5.20%) (Supplementary Figure 4.4). Further the growth of neuronal cells during *in vitro* differentiation showed higher growth potential of differentiating NPCs on DMS as compared to the control (Supplementary Figure 4.5). As described in our earlier studies (Vishwakarma et al., 2013), this protocol was followed to obtain more number of neurons as compared to astrocytes and oligodendrocytes to generate implantable neuronal construct for application in SCI repair wherein more number of neurons are desired as compared to glial cells. Hence, existence of large number of neurological cells was identified by staining with β-tubulin III which confirmed large number of well-organized and aligned differentiated neuronal cells on 3D-natural DMS. The expression of green fluorescence protein (GFP) by neuronal cells and axonal process of neurons stained with β-tubulin III showed coalesced into discrete 3D-DM architecture spanning the cellular population directly adhered to the scaffold (Supplementary Figure 4.6). The core difference between the neuronal expansion in 2D and 3D-meningeal scaffolds has been represented in Figure 4A. We observed simple and reductant neuronal cell expansion in 2D (Figure 4B) whereas neuronal cells grown on DMS showed highly organized growth with intact axonal tracts on DMS (Figures 4C,D) which provides both structural and biochemical cues. To demonstrate this phenomenon we used high resolution microscopy which revealed that DMS supports the generation of long neuronal axons first termed as pioneer axon which provides the cues for generating follower neurons by forming new synapses. This event is crucial to provide cues for the development of new long-distance follower axonal tract and host axons during regeneration process (Supplementary Figures 4.6, 4.7). The brief mechanism of this event is summarized by schematic representation in Figure 4E. Further characterization of these neurological features and cellular connectivity, microtubule-associated protein-2 (MAP-2) antibody staining was performed at day 21 in cultured neuronal cells on DMS (Figure 4F). MAP-2 staining revealed long-distance axonal development with new synapse formation among the neurological cells grown on DMS. Growth factor analysis required for neuronal regeneration within the MNC showed significantly higher levels than control at day 14 and day 21 for VEGF (Figure 4G), BDNF (Figure 4H) and NGF (Figure 4I) (^***^p < 0.0001). The levels of these cytokines were significantly higher at day 21 as compared to day 14 for MNC. Notably, at all time points MNC showed better levels of cytokine profile as compared to control (2D-culture) condition. Furthermore, mechanical strength of MNC was determined by measuring tensile strength which showed almost similar level to FM at day 14 and day 21 (Supplementary Figure 4.8).

### Assessment of functionality of MNC using pre and post-synaptic markers

The functional assessment of MNC was determined using immunohistochemical staining with pre-synaptic marker Synapsis-1 and post-synaptic marker Post Synaptic Density 95 kDa protein (PSD-95) along with neuronal structural marker MAP-2. This analysis showed highly increased positive staining of pre-synaptic granules for Synapsisn-1 with punctate distribution in axonal regions of the neuronal cells in MNC (Figure 4J). Similarly, PSD-95 staining also showed small punctae along with neuronal processes (Figure 4K).

### Molecular analysis for the expression of structural and functional gene transcripts within MNC

**Figure 5:**
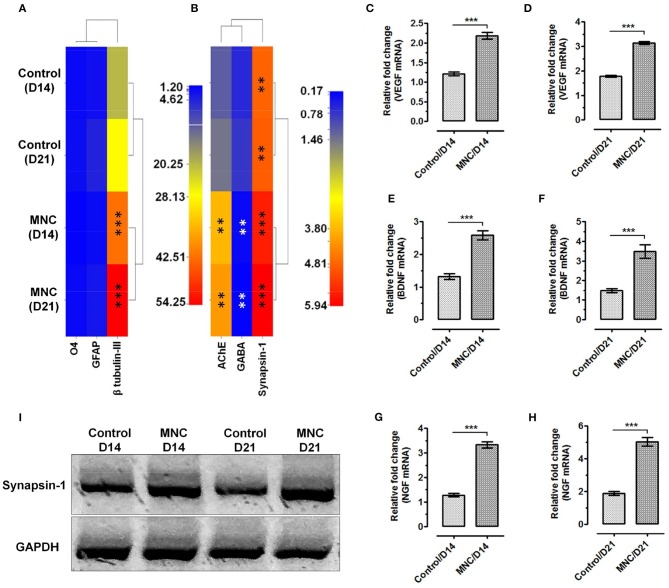
Heat map showing the gene expression levels of **(A)** neural lineage markers; β-tubulin III for neurons, GFAP for astrocytes and O4 for oligodendrocytes. Significantly enhanced expression of neuronal cell specific marker β-tubulin III was observed in cells cultured on DMS as compared to the control at day 14 and day 21 (^***^p < 0.0001) **(B)** The functional assessment of AChE and Synapsin-1 showed significantly higher level of mRNA expression as compared with the control condition. Whereas, significant down regulation was observed for GABA in neuronal cells grown on DMS as compared with the control (^**^p < 0.001). The expression level of VEGF at **(C)** day 14 and **(D)** day 21 showed significantly higher expression than control condition (^***^p < 0.0001). Similarly, the expression levels of BDNF **(E,F)** and NGF **(G,H)** was found significantly upregulated with respect to control (^***^p < 0.0001). **(I)** Western blot analysis showing induced expression of neuronal functional marker Synapsin-1 at protein levels in MNC.

Molecular analysis using relative mRNA expression was also performed to determine the transcriptional activation of structural and functional gene transcripts within the MNC as compared to control condition. Structural markers were used to investigate the percentage of lineage differentiation. Analysis of neuronal cell specific marker β-tubulin III showed >20-fold upregulated expression in MNC at day 14 and day 21 as compared to the cells cultured on fibronectin (control condition) (^***^p < 0.0001). Whereas, the expression of glial markers such as glial fibrillary acidic protein (GFAP) specific to astrocytes and O4 (specific to oligodendrocytes) showed very least expression when compared with β-tubulin III in both control condition and MNC (Figure 5A).

Furthermore, expression analysis of neuronal functional makers such as acetylcholinesterase (AChE), an enzyme which catalyzes the breakdown of acetylcholine and some other choline esters that function as neurotransmitters, gamma-aminobutyric acid (GABA), an inhibitor of neurotransmitter signaling and neuronal signaling molecule synapsin-1 which is present in the nerve terminal of axons and more specifically in the membranes of synaptic vesicles was performed to depict the neurological functions within the engineered MNC. The mRNA expression levels of AChE was found significantly higher in MNC at day 14 (^**^p < 0.001) and day 21 (^**^p < 0.001) as compared to control condition (Figure 5B). Similarly, expression levels of synapsin-1 was found significantly higher within MNC than control (^***^p < 0.0001). However, the expression of GABA mRNA was significantly downregulated in MNC as compared to control at both time points (^**^p < 0.001) which represents the enhanced neurological signaling.

In addition to the structural and functional markers, expression analysis of neuronal growth factors responsible for neurological cell survival and function were also assessed using relative mRNA quantification. More specifically, the expression levels of neuronal growth factors specific mRNA such as VEGF (Figures 5C,D), BDNF (Figures 5E,F), and NGF (Figures 5G,H) were found to express significantly higher levels within MNC as compared to control condition at both day 14 and day 21 (^***^p < 0.0001).

### Protein level analysis of neuronal function

Post-transcript verification, engineered human MNC was evaluated for the expression of functional neuronal marker synapsin-1 at protein level using western-blot and immunofluorescence analysis. The western-blot analysis revealed significantly higher synapsin-1 protein expression at day 14 and day 21 as compared to the control condition (Figure 5I).

### Bioengineered MNC shows *in vivo* bio/immune compatibility

**Figure 6:**
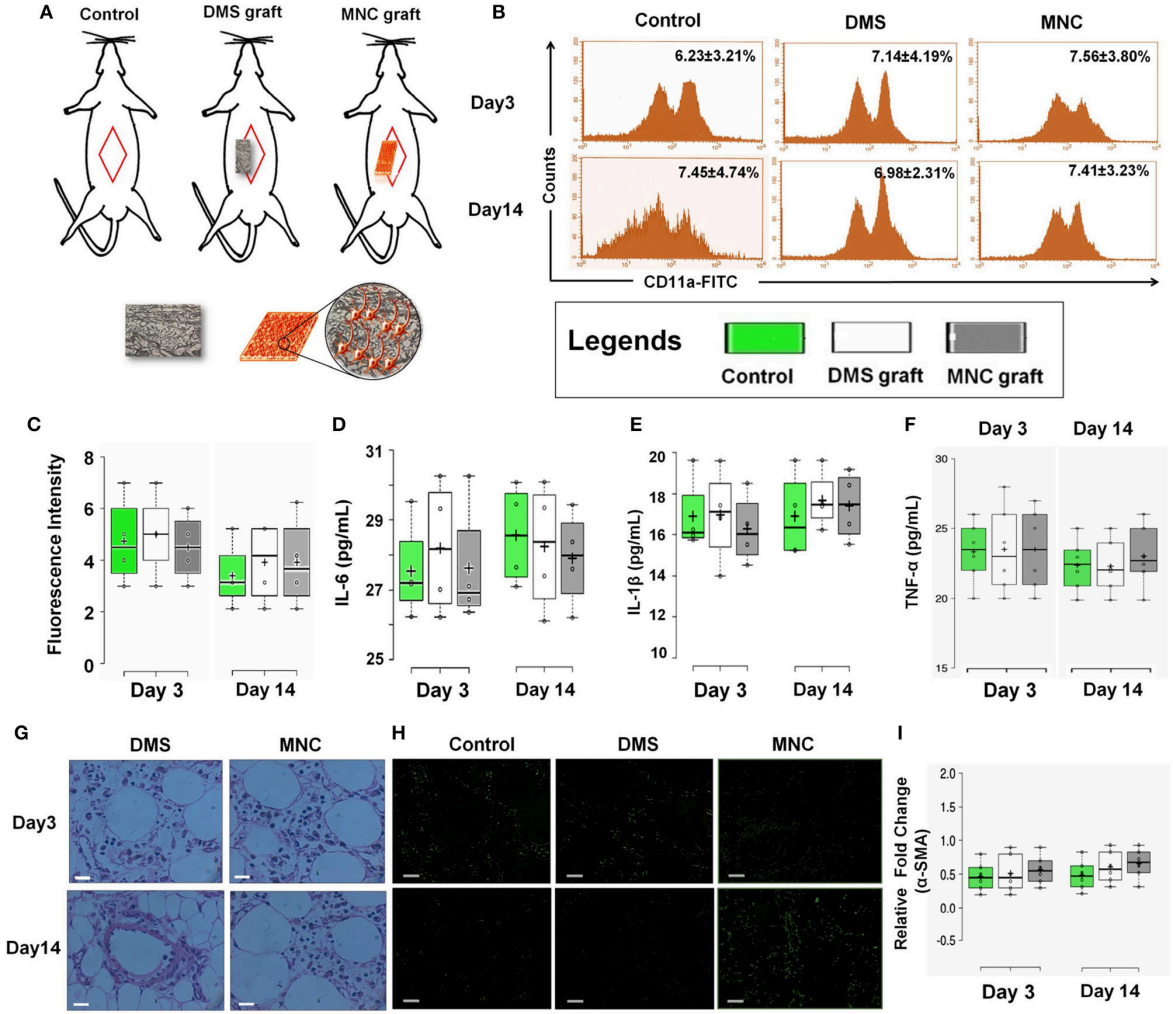
**(A)** Schematic representation showing *in vivo* intra-peritoneal transplantation of DMS and MNC for biocompatibility studies. **(B)** Histographs of CD11a in blood cells using flow cytometry revealed no significant change in cells expressing CD11a antigen at post-transplantation day 3 and 14 (*p* > 0.05). **(C)** Plots showing no significant change in fluorescence intensity of CD11a expression levels at post-transplantation day 3 and day 14 among control, DMS, and MMC. **(D–F)** The level of inflammatory cytokines in serum of rats withdrawn at post-transplantation day 3 and 14. No significant change was observed in both the groups [i.e., rats receiving (i) DMS and (ii) MNC implants (*p* > 0.05)]. **(G)** H&E staining of surrounding tissues of retrieved implants at day 3 and day 14 showing no sign of migrated/non-migrated inflammatory cells either in DMS or in MNC post-transplantation. **(H)** Immunofluorescence staining of surrounding tissues of retrieved DMS and MNC implants at day 3 and 14 showing no significant difference in fibrotic response as evident by α-SMA as compared to the control. **(I)** No change in the expression levels of fibrotic marker α-SMA was observed after day 3 and day 14 post-transplantation into the peritoneal tissues containing the graft.

One of the crucial aspects of any biological construct for tissue engineering applications is that it must be bio/immune compatible wherein cells must adhere efficiently, proliferate and function before laying down the new matrix. Hence, to check for the *in vivo* applicability of engineered MNC, *in vivo* intra-peritoneal transplantation was performed within Wister rats (Figure 6A and Supplementary Figure 6.1). Assessment of acute inflammatory response was determined using inflammatory cytokines analysis, changes in serum and hematological parameters, histological changes, CD11a staining and α-SMA analysis for fibrotic response. CD11a has been proposed to play crucial role in regulation of T-cells differentiation and central memory development in response to acute phase injuries. Hence, immunophenotypic analysis of CD11a was identified for DMS and MNC post-intraperitoneal transplantation. The flow cytometry analysis of CD11a expressing cells post-transplantation didn't show significant difference with the control groups which reveals absence or less immunological response against DMS and MNC by the host immunological system (Figures 6B,C). Almost similar fluorescence intensity of CD11a stained cells in host blood were observed for both DMS and MNC with respect to control at day 3 and day 14 post-transplantation (Figure 6C). Further, quantification of pro-inflammatory cytokines such as Interleukin-6 (IL-6) and Interleukin-1β (IL-1β) and acute phase cytokine TNF-α showed no significant change in serum levels at day 3 and day 14 post-transplantation for both DMS as well as MNC with respect to control (Figures 6D–F and Supplementary Figure 6.2).

H&E staining of surrounding tissues around the implanted DMS and MNC didn't show any sort of structural changes at day 3 and day 14 post-transplantation (Figure 6G). The peritoneal vascular bed was highly intact with normal distribution of cells within the tissues. Furthermore, α-SMA immunostaining of surrounding peritoneal tissues around the DMS and MNC at day 3 and day 14 post-transplantation didn't show any sign of fibrotic response in from of fibrotic tissue disposition around the graft which was further confirmed by expression levels of α-SMA transcripts in comparison to control (Figures 6H,I). Similarly, no significant change in hematological, liver and renal function parameters were observed at day 3 and day 14 post-transplantation for both DMS as well as MNC (Supplementary Tables 1–5). Histological investigations of vital organs post-transplantation of DMS and MNC also supported these finding which reveals the safer *in vivo* applicability of both types of engineered constructs (Supplementary Figures 6.3–6.6).

### Structural and functional characteristics of retrieved MNC

**Figure 7:**
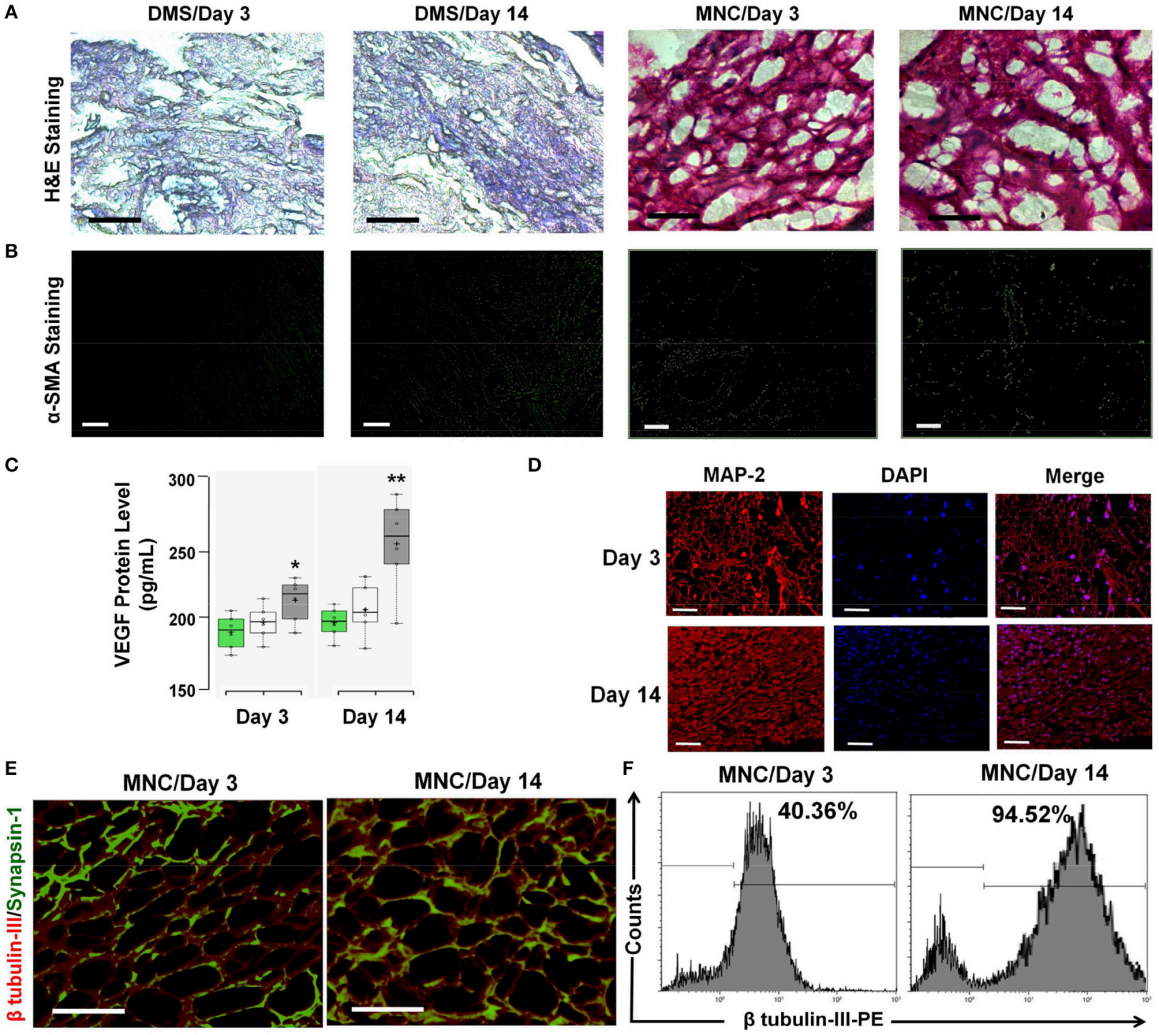
**(A)** The infiltration of immunological cells in surrounding tissues of the DMS and MNC implant was not observed as demonstrated by the H&E staining of peritoneal tissues. **(B)** α-SMA staining of DMS and MNC implants (retrieved after day 3 and day 14) didn't show significant positivity for fibrotic reactions. **(C)** VEGF levels within implanted grafts showed significantly higher amount at both the time points, however the levels were significantly higher at day 14 as compared to day 3 in MNC as compared to other two groups (^*^p < 0.01, ^**^p < 0.001). **(D)** MAP-2 staining (red) along with DAPI (blue) showing arrangement and connectivity of neuronal cells in more organized manner similar to the natural tissues retrieved MNC at day 3 and day 14. **(E)** Immunofluoscence analysis of neuronal functional marker Synapsin-1 (green) along with β tubulin-III (red) within engineered MNC showing structural and functional significance. **(F)** Immunophenotypic analysis for the presence of neuronal cells in retrieved MNC implants showed significantly high percentage of neurons expressing β-tubulin III at day 14 as compared to day 3.

The structural changes and accumulation of immunological cells infiltration in implanted DMS and MNC was estimated using H&E staining. The H&E analysis revealed absence of immunological cells infiltration in the implanted DMS and MNC within the retrieved grafts at day 3 and day 14 post-transplantation (Figure 7A). The fibrotic response generated by the host system was evaluated to confer the suitability of the MNC for long-term support for *in vivo* neural tissue regeneration. The fibrotic response against MNC after intra-peritoneal transplantation was evaluated using α-SMA antibody staining in retrieved MNC implants at day 3 and 14 post-transplantation. The qualitative investigation with immunocytochemical staining showed very weak staining for α-SMA in retrieved implants (Figure 7B and Supplementary Figure 7.1). Furthermore, neo-vascularization in peritoneal tissue grafts was determined by the enhanced expression levels of VEGF in the implants at day 3 and day 14. The protein quantification of VEGF levels within implanted grafts showed significantly higher amount at both the time points, however the levels were significantly higher at day 14 as compared to day 3 in MNC as compared to other two groups (Figure 7C). The structural organization of neurological cells was determined by immunofluorescence staining using MAP-2 antibody of MNC retrieved at day 3 and day 14 post-staining (Figure 7D). MAP-2 staining of neuronal cells within MNC at day 3 and 14 showed more organized cell expansion with long axons and well developed dendrites. Furthermore, high resolution detailed analysis of retrieved MNC at day 3 and day 14 using β-tubulin III revealed existence of large number of neuronal cells expressing β-tubulin III within MNC in similar fashion with respect to pre-transplantation. Additionally, the functional response of retrieved MNC was verified through synapsin-1 analysis which revealed enhanced neuronal signaling in form of enhanced synapsin-1 expression with punctuate distribution along-side of axonal networks throughout the MNC. Formation of newer synapses was more evident at day 14 as compared to day 3 (Figure 7E). The percentage neurological cells within MNC post-transplantation were determined using flow cytometry analysis for cells expressing β-tubulin III cytoskeleton protein (Figure 7F). This analysis showed significantly higher percentage of cells expressing β-tubulin III at day 14 as compared to day 3 post-transplantation representing higher levels of cell survival, engraftment and differentiation with increasing the time of transplantation. Collectively, these bio and immune compatibility assays revealed that MNC do not elicit systemic immunological responses and provides more suitable neuronal grafts with enhanced neuronal cell survival and function.

## Discussion

The present study describes preparation and use of DMS to engineer human MNC encompassing well organized functional neurological cells. The DMS developed herein preserves intact 3D-architecture, native ECM proteins, biological cues and half of the mechanical strength which is essential for long-term survival and function of neurological cells. Furthermore, specific organization of ECM proteins within the DMS provides guided hospitable microenvironment for more synchronized cellular engraftment and function. Several other studies have attempted to use such bioengineering approaches using different kinds of biological and synthetic scaffolds, however reported to provide insufficiency of desired mechanical strength, reduced growth factors signaling and least regenerative support (Nomura et al., 2006; Zahir et al., 2008; Wang et al., 2012; Shrestha et al., 2014; Libro et al., 2017; Liu et al., 2017).

DMS developed herein passes sufficient mechanical strength endowed with several crucial neural regenerative factors which are very much essential for mechanical as well as biological support to regenerate damaged neurological tissues in conditions like SCI. Owing to these peculiar characteristics, we adopted a unique, simple, fast, and highly reproducible method to generate DMS while retaining 3D-architecture, ECM, intact vasculature, sufficient mechanical strength (almost 50%) and crucial neurological regenerative factors such as VEGF, b-FGF, BDNF, and NGF.

GAGs are one of the most important components of innate ECM which provides not only a structural quality to the tissue but also confer organ-specific functional properties with a direct involvement in cell migration, proliferation, and differentiation. Moreover, GAGs' role in morphogenesis, angiogenesis, immune response, cellular homeostasis, structural resistance to tension, and compression has been studied in depth (Esko et al., 2009). GAGs have the capacity to assemble protein-protein complexes including growth factor receptors (for instance, TGF-beta, VEGF, EGF family members, and PDGF) both in extracellular environment and on cellular surface. Therefore, lack of adequate GAGs in ECM scaffolds may impair the ability to regenerate the cellular compartment. Although GAG content close to that of the natural tissue should be beneficial for cell signaling, some of the earlier studies have indicated that a partial reduction in GAG content might be beneficial to create a less dense matrix that allows cell infiltration and migration (Mohan et al., 2014). In the present study, 50% decrease in GAGs may allow more favorable engraftment and expansion site for cells to interact with ECM of DMS (Figures 2C,D). Decrease in 50% of GAGs content was not found to generate any adverse effect for cellular expansion and differentiation on DMS. However, slight decrease in mechanical properties of DMS was observed (50%) when compared to the natural tissue which was not found to affect the structural and functional properties of engineered MNC. This might be due to preservation of other tissue specific ECM proteins such as fibronectin (Figure 2A) and laminin (Figure 2B). Fibronectin is a large glycoprotein which is essential for supporting cellular adhesion through binding with integrins. It also supports axonal regeneration and neurites outgrowth (Leiss et al., 2008; Tongea et al., 2012). Laminin is another key component of ECM which facilitates interaction of neuronal cells and provides essential attachment points which enables axonal extension and exert forces on ECM (Selak et al., 1985; Varnum-Finney and Reichardt, 1994).

Furthermore, the decellularization process followed in this study to produce DMS revealed preservation of several crucial neurotrophins (Figures 3D–G) which are one of the most important signaling molecules involved in neuronal development and guidance. Among various kinds of neurotrophins, preservation of BDNF, GDNF, and NGF signifies importance of DMS for generating suitable neurological constructs for regenerative applications. More specifically, we have observed ~50% decrease in BDNF (Figure 3F) and NGF (Figure 3G) contents after complete decellularization of meningeal tissues, however no significant change was observed for VEGF (Figure 3D) and bFGF (Figure 3E) as compared to fresh meningeal tissues. The major reason for higher level retention of VEGF and bFGF within DMS is dependent on their stronger binding affinity with meningeal ECM (Caralt et al., 2016) while BDNF and NGF are secreted from neuronal cells and has relatively lower binding affinity with ECM. Furthermore, VEGF and bFGF are secreted from cells other than neuronal lineage and are essential or cell to matrix interaction and adherence. Whereas, BDNF and NGF are mostly involved in neuronal cell survival and function. NGF has also been found to promote axonal growth of neurological cells through activation of their corresponding tyrosine kinase (Trk) receptors (Tsoulfas et al., 1993), whereas BDNF is known to enhance survival and promote axon growth from retinal ganglion cells and hippocampal neurons via its receptor, TrkB (Soppet et al., 1991). Studies have also demonstrated that bFGF-2 influences neurons, schwann cells, and oligodendrocytes for axonal growth and remyelination following injury (Jungnickel et al., 2006; Wang et al., 2013). Furthermore VEGF, an important cytokine preserved in DMS can improve axonal outgrowth through VEGF receptor signaling (Park et al., 2016). In addition to these neurotrophins, integrity of meningeal vasculature post-decellularization provides support for increased supply of oxygen and nutrients for enhanced cell survival and function (Figures 3A–C). Thus, existence of preserved mechanical strength, ECM proteins, vasculature, and neurotrophins within the DMS depicts the usability of such biological scaffolds for developing suitable neurological constructs for regenerative application in conditions like SCI.

Based on the above findings, hNPCs were differentiated on DMS to develop MNC for regenerative applications in SCI. The practical advantages of DMS was analyzed during neuronal differentiation for cell viability, proliferation efficiency, directed axonal extension and formation of new synapses. In view of recently published successful studies on regenerating SCI, wherein human neural stem cells (Shetty and Hattiangady, 2016; Liu et al., 2017; Rosenzweig et al., 2018) were used to obtain maximum benefit for providing better support and induce endogenous regenerative processes. In present study, we used hNPCs to generate a proof of concept for developing better biological neural constructs for proposed application in SCI. The use of hNPCs derived from fetal SVZ may be replaced with neurological cells derived from human mesenchymal stem cells (MSCs) or induced pluripotent stem cells (iPSCs) in future which may avoid ethical concerns on the extensive use of such biomedical implants. However, the use of MSCs provides only immunomodulatory effect and its support in spinal cord to bridge the gap by trans-differentiating into neurological cells are limited and have showed very less success. Furthermore, use of iPSCs to generate human neurological cells for clinical application is limited due to very high level of genetic instability and tumorigenicity. Hence, in this study, our major aim for developing biomimetic neurological implants using human fetal SVZ derived NPCs to generate suitable neurological construct which can be used predominantly as institutional-based product rather off shell for the treatment of SCI patients. This particular approach provides key advantages for producing enriched neuronal constructs combined with natural biological scaffolds which are well tolerated by the host with high survival rate as demonstrated elsewhere (Wernig et al., 2004; Iwata et al., 2006).

Furthermore, *in vitro* modeling of neuronal cell growth on DMS could provide better platform for understanding regenerative mechanisms in SCI pathophysiology. Another important aspect of spinal cord tissue regeneration is to bridge the long-gaps formed between neurons due to injury. In view of this, very limited success has been demonstrated. Hence, we followed the growth pattern of neuronal cells on DMS (Figure 4). This particular investigation revealed utilization of preserved scaffold plasticity in directed guidance for arrangement and physical wiring of well-connected long axonal tracts of neurological cells (Figures 4F,J,K). This finding reveals the utilization of engineered MNC to sense and respond to local injuries at the transplantation site as described elsewhere (Colicos and Syed, 2006). The generation of well-connected long-axonal tracts in follower and pioneer neurons (Figure 4) suggests that these bioengineered tissue constructs could be transplanted directly to rebuilt the damaged axonal tracts and activate the host neuronal network to fasten the regenerative processes (Mukhatyar et al., 2009). The natural organoid model described herein as engineered MNC provides closer interaction of cells with ECM which leads to more organized biomimetic spatial patterning and cytoarchitecture formation as compared with the 2D cultures where cells exhibit polarity and neuronal growth form is irregular (Figure 4A). The engineered MNC showed significantly higher levels of VEGF, BDNF, and NGF representing better survival and function of the cells within MNC (Figures 4G–I). The pre and post-synaptic neuronal signaling was observed in connecting well developed neuronal axons identified using Synapsin-1 and PSD-95 antibody staining of MNC (Figures 4J,K). These observations revealed that axonal connectomes formed on DMS are arranged in linear fashion with newly developed synapses and functional activities required for supporting loss of function at injury site.

The gene expression analysis revealed enhanced expression of structural and functional molecular regulators in neurological cells cultured on DMS. Analysis of structural transcripts showed significantly higher expression of β-tubulin III demonstrating large percentage population of neuronal cells within MNC as compared with the glial cells which is a positive factor for application of engineered MNC in SCI regeneration (Figure 5A). More specifically AChE, an enzyme that catalyzes breakdown of acetylcholine and some other choline esters which function as neurotransmitter, GABA, an inhibitor of neurotransmitter signaling and Synapsin-1 which is a component of the vesicle that contains neurotransmitters expression levels were analyzed within MNC (Figure 5B). The analysis of these functional neuronal transcripts showed significantly enhanced expression levels of AChE and Synapsis-1 in MNC as compared to 2D culture system revealing the fact that neurological cell within MNC encompasses better molecular level of functional response than 2D-culture system. Furthermore, significantly reduced expression levels of GABA transcript showed induced neuronal excitability within MNC. The enhanced functional activities and neuronal cell survival was also supported by the finding of significantly increased expression levels of neurotrophins such as VEGF, BDNF, and NGF (Figures 5C–H). Moreover, protein level analysis of neurotransmitter signaling molecule synapsin-1 was also performed using western-blot analysis for MNC (Figure 5I). This analysis revealed that neurological cells within the MNC have better functional response as compared to control condition after day 14 and day 21. However, at day 21 neuronal cells on MNC showed higher level of synapsin-1 synthesis as compared to day 14 which signifies that the engineered MNC retains its higher level of functional activity in long-term.

For future successful *in vivo* application of such MNC, immune compatibility studies are of utmost important. These MNC should be pliable, harmless to the surrounding and adherent *in vivo* tissues and could resist its mechanical collapse during transplantation which may lead to necrosis and inflammation at the site. Allografts have been considered gold standards for nerve tissue reconstruction; however the major drawback remains the use of immunosuppressants during transplantation and severe life-threatening side-effects. Nerve autografts are the other alternative of allograft which provides better result, however loss of donor site function and morbidity, need for a secondary surgery, limited availability and structural differences between donor and recipient grafts poses major challenges for their wide applicability (Mukhatyar et al., 2009; Jiang et al., 2010). Hence, to depict the immunological tolerance of developed MNC intra-peritoneal transplantation was performed in Wister rats wherein MNC do not elicit systemic inflammatory response or harm to the surrounding tissues (Figure 6). During bio/immune compatibility studies, we observed that both DMS and MNC do not elicit the fibrotic response post-transplantation. All the animals survived up to entire duration of the study (i.e., 14 days post-transplantation) which revealed that these constructs are immune-compatible.

Furthermore, studies on the retention of mechanical strength and histological staining showed maintenance of normal architecture after retrieval while retaining its almost similar tensile strength (Supplementary Figure 4.8). The grafts were also intact with well-developed neuronal axons within MNC. Along with the structural features, functional response of the retrieved MNC grafts was also significantly preserved (Figure 7E). This particular functional phenotype may be well-suited for enhanced regenerative mechanisms at the lesion-site in SCI. This promising strategy for developing immunologically tolerable MNC may be simultaneously capable of physical restoration of damaged axonal connections, addressing the neuronal cell replacement and neuronal circuit modulation which may challenge the current quo of hardware-based neuromodulation (Bradbury et al., 2002; Borisoff et al., 2003; Jain et al., 2004; Yoshioka et al., 2007). Further pre-clinical testing and optimization of this approach in animal models will likely provide more authentic road map for disclosing the potential of such biomimetic neural tissue engineering approaches for future clinical applicability.

## Conclusion

The DMS developed in this study retains its natural ECM proteins, 3D-architecture, sufficient mechanical strength, and biological cues in terms of active molecules and growth factors which offers prominent advantages over the synthetic scaffolds where these characteristics needs to be produced artificially by adopting various fabrication strategies that later introduces complications with regard to biocompatibility and further pre-clinical and clinical applicability. Furthermore, these 3D-DMS encourages the alignment and directional growth of neuronal axons within the MNC which is immunologically tolerable *in vivo* and preserves its structural and functional characteristics post-transplantation. In conclusion, engineered human MNC using DMS platform may serve as one of the potential option to investigate regenerative potential in SCI models since they offer sufficient mechanical properties and biomimetic niche for regenerating new neuronal connectomes without eliciting systemic inflammatory response into the host tissues.

## Materials and methods

### Chemicals and reagents

All chemicals described in the experiments were purchased from Sigma-Aldrich (St. Louis, MO), Thermo Scientific (USA), Abcam and R&D system. Cell culture reagents such as Complete Neural proliferation (#05751) and Complete Neural Differentiation Medium (#08500) were purchased from Stem Cell Technologies (Canada). Epidermal growth factor (EGF, #236-EG), and basic fibroblast growth factor (bFGF, 233-FB-500) supplements were purchased from R&D System Inc. Minneapolis, USA. Human CD133 MicroBead Kit was purchased from Miltenyi (130-097-049, Miltenyi Biotec Inc. USA). Dulbecco's Phosphate buffer Saline (D-PBS) was purchased from Invitrogen (#14190-250, USA). Fibronectin coated coverslips (GG-12-fibronectin) were purchased from NeuVitro, USA. Glycosaminoglycans (GAGs) ELISA Kit (#6022) was purchased from Chondrex, Inc. Redmond, WA. Alcian blue stain (B8438-250ML), vascular endothelial growth factor (VEGF, RAB0507-1KT), fibroblast growth factor (FGF, RAB0182-1KT), human nerve growth factor (NGF, RAB0380-1KT), and human brain derived neurotrophic factor (BDNF, RAB0026-1KT) ELISA Kits were purchased from Sigma Aldrich, USA. Human IL-6 (D6050) and IL-1β (DLB50) ELISA kits were purchased from R&D System Inc. Minneapolis, USA. Antibodies used in this study were purchased as follows: Human α-SMA (ab5694, Abcam, USA, 1:100) and β-tubulin III (ab78078, Abcam, USA, 1:500) from Abcam, UK. Synapsin I (NB300-104, 1:500) and PSD-95 (6G6-1C9, 1:250) antibodies from Novus Biologicals, Southpark Way, A-8, Littleton, CO 80120. Anti-MAP2 antibody (M9942, 1:500) from Sigma Aldrich, USA, Laminin (MAB2066, R&D Systems, India, 1:50), Fibronectin (AF1918-SP, 1:100), and anti-hNestin (IC1259P, Lot No. LML0311041, 1:50) antibodies were purchased from R&D System, USA. CD11a-PE antibody (101107, 1:10) was purchased from Biolegend, USA.

### Procurement of human brain meninges

The discarded human brain meninges (*n* = 5) were obtained from the neurosurgery ward of Owaisi Hospital, Hyderabad after taking ethics approval from the Institutional Ethics Committee of Deccan College of Medical Sciences, Hyderabad. The study was conducted as per the ethical and regulatory guidelines. Signed informed consent forms were obtained from the parents before collecting the meningeal tissues. The collected meningeal tissues were washed twice with 1X Phosphate buffer saline (PBS) supplemented with 1X antibiotic and antimycotic solutions and sliced into pieces (3–4 cm^2^ each) and used for decellularized scaffolds preparation.

### Decellularized scaffolds preparation using human brain meninges

Each slice of human brain meninges was taken and spread uniformly on normal glass cover slips. Afterwards, cover slips with intact meningeal tissues were incubated with gradients of decellularization reagents in shaking incubator at 120 rpm and 37°C temperature. Initial decellularization was performed by incubating meningeal tissues spread on glass coverslips with 1X PBS supplemented with 0.05% Tween-20 (P9416, Sigma Aldrich, USA) for 10 min. Second incubation was performed with 0.1% SDS (436143, Sigma Aldrich, USA) diluted in 1X PBS for 10 min and the final incubation was done in 0.5% SDS for a total of 240 min until the removal of entire cell components. The DMS were then washed thrice with 1X PBS to remove SDS and other chemicals retained inside the scaffold. The decellularization procedures was repeated at least 10 times to improve the reproducibility of the method.

### Microscopic analysis

Microscopic observation was performed to identify the structural analysis before and after decellularization of meningeal tissues at different time points. Briefly, meningeal tissues before and after decellularization for 30, 60, 120, and 240 min were observed under 10X magnification of light microscope (Car Zeiss, Germany). The existence of cellular components and ECM was visualized to determine the extent of decellularization process in time dependent manner.

### Histological and immunohistochemical analysis

Meningeal tissues before and after decellularization for 30, 60, 120, and 240 min were fixed in 4% paraformaldehyde (PFA, P6148, Sigma Aldrich, USA) solution at room temperature for 15–20 min and processed in a tissue processor. Following to fixation, tissues were embedded in paraffin blocks using standard protocol. Three to five micrometers thin sections were prepared and stained with hematoxylin and eosin (H&E, NC0510871, Fischer Scientific, UK) stain and alcian blue (B8438-250ML, Sigma Aldrich, USA) to identify the presence of residual nuclear content and intact ECM of post decellularization. For immunohistochemical analysis, sections were deparaffinized and hydrated. Antigen retrieval was performed using SDS to expose epitope more effectively. Blocking was performed using 1% bovine serum albumin (BSA) solution for 30 min at 37°C temperature and then incubated with primary antibodies for overnight at 4°C. Following to this incubation, washing was done thrice to remove unbound antibodies and further incubated with secondary antibodies for 60 min at room temperature. Counterstaining was done using 4,6-diamidino-2-phenylindole, dihydrochloride (DAPI, D9542, Sigma Aldrich, USA). Sections were viewed and images were captured under an inverted fluorescence microscopy (Car Zeiss, Germany).

### Evaluation of residual nucleic acid content

The lysate of meningeal tissues before and after decellularization for 30, 60, 90, 120, 150, 180, 210, and 240 min was prepared through enzymatic digestion using papain solution (0.1%, P4762, Sigma Aldrich, USA), 1 mM EDTA (798681, Sigma Aldrich, USA), 7.0 mM cystein (C7352, Sigma Aldrich, USA), and 1M NaCl (S9888, Sigma Aldrich, USA) in 1X PBS at 60°C for 48 h within an incubator shaker. The residual nucleic acid content in lysate was quantified by spectrophotometric analysis at 260 and 280 nm.

### Quantification of residual glycosaminoglycans

Quantification of glycosaminoglycans (GAGs) in fresh and DMS after 30, 60, 120, and 240 min was performed as per the manufacturer's instructions as provided in GAGs ELISA Kit (#6022, Chondrex, Inc). Briefly, samples were prepared by complete solubilization after enzymatic digestion. Different concentrations of solutions were prepared for creating standard curve. Along with the controls, test sample lysates were added in microplates in triplicates and sample absorbance was obtained at 530 nm using a Microplate Reader (Biorad). The μg/mg concentration of GAGs in test samples was calculated using regression analysis and multiplied it by the sample dilution factor to obtain the GAG concentration in the original test samples.

### Analysis of tensile strength

To determine the mechanical integrity, tensile strength analysis of meningeal scaffolds before and after decellularization was performed according to ASTM D1708 (Kakabadze et al., 2016). Prior to this analysis DMS were completely hydrated in 0.9% saline. The tensile strength was estimated by pulling the samples at 50 mm/min of failure using a mechanical test stand and reported in MPa of sample width.

### Microstructure analysis of DMS using scanning electron microscopy

For microstructure analysis of DMS, samples were freeze-fractured and fixed with 2.5% gluteraldehyde in PBS (pH 7). Following to fixation process, samples were washed twice with 1X PBS and further fixed with 1% osmium tetraoxide. After completing the fixation process, samples were rinsed twice with distilled water and dehydrated using successive ethanol treatments prior to critical-point drying with liquid CO_2_. Samples were directly mounted for surface visualization whereas cross sections were prepared before mounting to identify clear vascular networks within DMS. Scanning electron microscopy (SEM) analysis was performed using JOEL-JSM 5600 SEM at RUSKA Lab's College of Veterinary Science, SVVU, Rajendranagar, Hyderabad, India as per the standard protocol (Bozzola and Russell, 1998).

### Analysis of retained cytokines in DMS

The amount of residual cytokines in DMS was identified by ELISA using VEGF (RAB0507), FGF (RAB0182), BDNF (RAB0026), and NGF (RAB0380) kits. Briefly, the soluble molecules were extracted from fresh as well as DMS after 30, 60, 120, and 240 min of decellularization using tissue extraction reagent (FNN0071, Invitrogen) and homogenized. Supernatants were collected after centrifuging the lysate at 10,000 rpm for 5 min. The quantification of VEGF, FGF, BDNF, and NGF cytokines was performed in lysate supernatant by ELISA analysis according to the manufacturer's instructions (Sigma Aldrich, USA). Each analysis was performed in triplicates in three independent experiments.

### Isolation, enrichment, and culture of human neural precursor cells on DMS

Human neural precursor cells (hNPCs) were obtained from cryopreserved human fetal subventricular zone (SVZ) tissues (*n* = 3, gestation age: 10 weeks). Briefly, SVZ tissues were minced and enzymatically digested using 0.03% collagenase enzyme at 37°C in a CO_2_ incubator for 15–20 min. Following to digestion, single cell suspension was prepared by filtering digested tissues through 40 μm cell strainer. Furthermore, enrichment of hNPCs in total isolated SVZ-derived cells was performed using magnetic activated cell sorting (MACS) with the help of prominin-1 stem cell specific magnetic beads. The cell viability of both prominin 1 positive and negative fraction was estimated using Trypan blue exclusion method and further verified by 3-(4,5-dimethylthiazol-2-yl)-2,5-diphenyltetrazolium bromide (MTT) reduction assay. Both the fractions were analyzed for their neurosphere development ability as described in our earlier studies (Vishwakarma et al., 2013, 2017, 2018). Immunofluorescence and molecular analysis was also performed to determine the percentage positive cells for neural precursor markers (such as Nestin) existing in neurospheres derived from both the fractions using anti-hNestin antibody (IC1259P, R&D System, USA) and specific oligonucleotide primers. Based on the observations, prominin-1 positively sorted cells were used for conducting further experiments in this study.

For culturing prominin-1 positive sorted cells, sizes of DMS were calculated and hNPCs were seeded in neural differentiation medium supplemented with Retinoic acid (RA) and 2% heat-inactivated human umbilical cord serum (UCS) as described in our earlier study (Vishwakarma et al., 2013). Briefly, committed hNPCs derived from developing neurospheres were dissociated into single cell suspension using enzymatic digestion and filtration through 40 μm cell strainer. Furthermore, NPCs were seeded at density of 1.5 × 10^5^ cells on per cm^2^ area of DMS. The neurogenic differentiation was triggered using mitogen free complete human differentiation supplements (#08500, Stem Cell Technologies, Canada) containing 0.05 μM RA and 2% human UCS. The use of human UCS was adopted to avoid xenogeneic supplements for future human applications. The neurogenic differentiation medium was replenished with fresh medium every after 3^rd^ day of culture. After 24 h of cell seeding MTT assay was performed as per the standard protocol to identify the cell adhesion and viability on DMS. Cells on fibronectin coated coverslips were used as control to compare the difference in cell adhesion and viability. In addition to this, MTT cell proliferation assay was performed at day 3, 7, 14, and 21 to identify the percentage cell growth as compared to the control condition.

### Microscopic observation and immunofluorescence analysis

Prominin-1 positively enriched hNPCs cultured on DMS were monitored continuously under inverted microscope and carefully observed the growth, survival and morphological changes at day 7, day 14, and day 21 in comparison to control condition post-neurogenic differentiation. The light microscopic images were captured in 10X magnification to identify the neurological networks. Characterization of neurological cells differentiated on fibronectin coated coverslips (control condition) and DMS was performed using β-tubulin III and microtubule associated protein (MAP-2, M9942, 1:500) antibody immunocytochemical staining at day 21. Furthermore, MAP-2 antibody staining along with pre-synaptic marker synapsin-1 (NB300-104, 1:500) and post-synaptic marker PSD-95 (6G6-1C9, 1:250) was performed in neurological cells grown on DMS. DAPI was used as counter stain to identify the cellular nuclei. Fluorescence images were captured and documented using Axiocam software (version 4.1.2) in inverted fluorescence microscopy (Carl Ziess, Germany).

### Quantification of specific growth factors in neurological cells grown on DMS

The amount of neuronal growth factors required for cell survival and function was quantified using specific ELISA kits for VEGF (RAB0507), BDNF (RAB0026), and NGF (RAB0380). Briefly, culture supernatants of neurological cells grown on DMS at day 14 and day 21 were collected and directly used as test samples for reading along with positive and negative controls as per the manufacturer's instructions (Sigma Aldrich, USA). Each analysis was performed in triplicates in two independent experiments to avoid technical errors. The quantity of VEGF, BDNF, and NGF in cells grown on DMS was compared with the control condition at day 14 and day 21.

### Gene expression analysis

To evaluate the gene expression levels in differentiating neurogenic cells on DMS, cells were harvested from the culture at day 14 and day 21. Total ribonucleic acid (RNA) was extracted using guanidium isothiocyanate (GITC) method and converted into complementary DNA (cDNA) by using oligodT primer and MMLV reverse transcriptase enzyme II (28025013, Thermo Scientific, USA). RT-qPCR was carried out using gene specific primers Nestin-forward CTGCTACCCTTGAGACACCTG and reverse GGGCTCTGATCTCTGCATCTAC, β-tubulin-III-forward CTCATGGACTGATTATGGACAGGAC and reverse GCAGGTCAGCAAAGAACTTATAGCC, glial fibrillary specific protein (GFAP)-forward CTGCGGCTCGATCAACTCA and reverse TCCAGCGACTCAATCTTCCTC, O4-forward CTACTGCTCTGGGTCCCAGG and reverse CTGCCACTGAACCGAGATGG, acetylcholine esterase (COLQ/AChE)-forward CTTCCTACGGGGAATCTGTGT and reverse CAATGGCGTTTTGGGTGTTC, Gamma-amino butyric acid (GABA)-forward ACGTCCGTGTCCAACAAGTC and reverse AAAGTCGAGGTCGTCGCAATG, Synapsin-1-forward AGTTCTTCGGAATGGGGTGAA and reverse CAAACTGCGGTAGTCTCCGTT, VEGF-forward GAGGAGCAGTTACGGTCTGTG and reverse TCCTTTCCTTAGCTGACACTTGT, BDNF-forward TAACGGCGGCAGACAAAAAGA and reverse TGCACTTGGTCTCGTAGAAGTAT, NGF-forward GGCAGACCCGCAACATTACT and reverse CACCACCGACCTCGAAGTC, and endogenous control Glyceraldehyde 3-phosphate dehydrogenase (GAPDH)-forward TGTGGGCATCAATGGATTTGG and reverse ACACCATGTATTCCGGGTCAAT in both controls (cells cultured on fibronectin coated surfaces) as well as on DMS using SYBR Green assay protocol. RT-qPCR condition was optimized to 95°C for 5 min for one cycle and 95°C for 30 s, 52–60°C for 30 s, and 72°C for 30 s for 40 cycles. A final melting curve analysis was performed to exclude the primer-dimers from the real amplification curves.

### Western blot of synapsin-1

Protein extraction from MNC at day 14 and day 21 post-neuronal cells differentiation was prepared using radioimmunoprecipitation assay buffer (chilled RIPA buffer). Each protein extract was diluted with denatured polyacrylamide gel electrophoresis (PAGE) sample buffer and heated at 70°C for 10 min. Twenty-five microliters of each protein extract were then loaded and subjected to electrophoresis in SDS Running Buffer with at 200 V for 25–30 min. Following separation, proteins were electro transferred onto 0.2 μm nitrocellulose membrane in 1X PAGE Transfer Buffer at 30 V for 60 min. Post protein transfer, membranes were blocked using 5% milk powder in 1X PBST and incubated for 60 min at room temperature with gentle shaking. Furthermore, 10 mL of anti-synapsin primary monoclonal antibody (1 μg/mL) in 5% milk with 1X PBST was added to the membrane and incubated at 4°C for overnight with gentle shaking. Unbound antibodies were washed trice with 1X PBST and 10 mL of secondary goat anti-rabbit IgG antibody conjugated to HRP (0.2 μg/mL, diluted in 1X PBST) was added and incubated 60 min at room temperature with gentle shaking. Post-incubation secondary antibody was washed twice with 1X PBST. Two milliliters of TMB membrane peroxidise substrate was pipette onto the membrane and incubated additionally for 5 min in dark. Sterile water was used to stop the reaction and color development was recorded digitally. GAPDH was used as endogeneous control to compare the expression of synapsin-1 with respect to GAPDH in same sample. The western blot analysis was performed three times in three different cohort experiments using same sample to confirm the results.

### Animal studies

All animals related experimental procedures used in this study were approved by the Institutional Animal Ethics Committee of Deccan College of Medical Sciences, Hyderabad. Experiments were conducted as per the relevant regulatory guidelines. A total of 24 animals (Wister rats, Gender: Male, Weight: 250 ± 25 g) were obtained from the National Institute of Nutrition (NIN), Hyderabad. The animal experimentation was conducted to identify the *in vivo* bio/immune compatibility of MNC (i.e., neuronal cells grown on DMS).

### Transplantation surgeries

During the operative procedures, animals were anesthetized by sub-cutaneous injection of Buprenorphine (0.05 mg/kg of body weight) along with 300 μl of saline (0.9%) to prevent dehydration. To expose the peritoneal lining, a 1–2cm incision was made in middle of the abdomen of each animal. Peritoneal wall was grabbed and further ~1 cm incision was done along with the lining of linea-alba. DMS (*n* = 8) and MNC (*n* = 8) were transplanted into the peritoneal cavity through incision using scalpel facing cells toward the peritoneal cavity. Incision was sealed with the help of absorbable sutures and skin was closed over the incision using clip and tissue glue. Animals without any transplant were considered as controls (*n* = 8).

### Assessment of hematological changes post-transplantation

One milliliter orbital sinus blood was collected in EDTA-coated tubes from each group of animal at day 3, day 7, and day 14 post-transplantation. After anesthetizing the skin around the head and neck was gently pulled to protrude the eyeballs slightly. A sterile hematocrit tube was inserted carefully into inner corner of each eye directing toward the midline. A gentle pressure was applied to the hematocrit tube to pierce the retro-orbital sinus. The blood flow was adjusted by keeping the tip of the tube down in assistance of gravity. After collecting the blood, hematocrit tube was removed and a gentle pressure was applied to the eye for 15–30 s to stop the blood flow. After achieving the homeostasis, antibiotic ophthalmic ointment was applied to the eye to prevent the risk of infections. All animals were transferred to the recovery cage and monitored continuously until it is fully awake and can walk normally. Each blood sample was aliquoted and used for the analysis of hematological parameters such as hemoglobin, platelets, hemocrit (HCT), white blood cells (WBCs), red blood cells (RBCs), Neutrophils, Basophils, Eosinophils, Monocytes, and Lymphocyte counts using hematological autoanalyzer (Pentra ES_60_, Horiba, France) with the help of specific kits.

### Analysis of biochemical parameters

Remaining blood was transferred in serum collection tubes coated with coagulation factors and incubated at room temperature for 1–2 h. Following to incubation, centrifugation was performed at 3,000 × g for 15 min at 4°C. Finally the serum was separated and used for the analysis of biochemical parameters such as liver function tests (SGPT, SGOT, ALP, total bilirubin, and albumin) and renal function parameters (urea and creatinine) using Biochemical Analyzer (Microlab 200, Merck). The analysis of each biochemical parameter was done by standard operating procedure as per the instructions of manufacturers.

### Immunophenotypic analysis of CD11a

The blood samples collected in EDTA-coated tubes from each animal was stored at room temperature and mixed by gentle agitation prior to use. One hundred microliters of blood was aliquoted and 5 μL of the CD11a-PE antibody (101107, Biolegend, USA, 1:10) was added to each tube. After mixing with the antibody, tubes were incubated for 30–60 min at 2–8°C. After incubation 2 mL of 1X RBC Lysis Buffer was added in each tube and mixed gently. Further samples were incubated in dark at room temperature for 10 min. Samples were centrifuged at 300–400 × g for 5 min at room temperature. Supernatant was aspirated and cells were washed once with 2 mL of Flow Cytometry Staining Buffer. Again centrifugation step was repeated and supernatant was aspirated. Finally the stained cell pellet was resuspended in 500 μL of Flow Cytometry Staining Buffer and analyzed using a Flow Cytometer. Unstained blood cells were used as isotype control.

### Serum cytokine ELISA

Changes in the serum levels of three important inflammatory cytokines Interleukin-6 (IL-6, D6050, R&D System), Interleukin-1β (IL-1β, DLB50, R&D System), and TNF-α (MTA00B, R&D System) was quantified by suing specific kits as per the manufacturer's instructions. The levels of these cytokines was determined in each group of rats at post-transplantation day 3, 7, and 14 which was further compared with the control groups to estimate the level of inflammatory response against the transplanted DMS and MNC.

### Organ histology

The presence of inflammation and damage in vital organs was identified by histological staining of harvested the rat organs at post-transplantation day 3 and 14. H&E staining was performed for each section of the surrounding peritoneal tissues and implanted DMS and MNC at day 3 and day 14 post-transplantation. The careful histological grading was done in blinded manner by an expert veterinary pathologist. Briefly, the liver inflammation and damage was scored in terms of histology activity index or Knodell score as described earlier (Batts and Ludwig, 1995; Brunt et al., 2009). Zero (0) score was given for no sign of inflammation, score 1–4 for minimal, 5–8 for mild, 9–12 for moderate, and 13–18 for the presence of severe inflammation in the tissue. Similarly the renal damage was scored by careful analysis of renal glomerular degeneration and renal tubule necrosis as described earlier (Gu et al., 2016). The lung damage was scored semi-quantitatively by modified protocol as earlier defined (Matute-Bello et al., 2011). The total lung inflammation was scored as sum of the total score of each parameter. Further the cardiac tissue damage was identified by the standard operating procedure described by Hölschermann et al. (1999). While the brain tissue damage was evaluated in different areas of the brain and expressed in terms of percentage damage as described earlier (Simmons et al., 2014).

### H&E and α-SMA staining of retrieved grafts

H&E staining was performed as described in earlier section to identify the infiltration of immunological cells in implanted grafts. Whereas, fibrotic response against the implants in Wister rats was identified using immunohistochemical staining using antibody specific to alfa-smooth muscle antigen (α-SMA). Furthermore, mRNA expression analysis of α-SMA was performed in cells harvested from retrieved implants at day 3 and day 14 post-transplantation in rat peritoneum. Briefly, total RNA was isolated and converted in cDNA which was used as template to perform relative quantification of gene expression using SYBR Green-based real-time quantitative assay. The comparative analysis for the expression levels of α-SMA in control, DMS and MNC was performed using fold change calculation with respect to endogenous control (i.e., GAPDH) as described in earlier section.

### VEGF quantification

To determine the neovascularization within the engrafted DMS and MNC post-intrperitoneal transplantation, quantification of vascular endothelial growth factor (VEGF) was performed using Mouse VEGF Immunoassay kit (MMV00, R&D System) as per the manufacturer's instructions. Lysates prepared from each implant along with surrounding host tissues were used for VEGF estimation and compared with the control. The amount of VEGF was expressed in pg/mL of the lysate used for estimation. Five samples of each group were used for VEGF estimation in three different experiments to enhance the reproducibility of the analysis.

### Immunohistochemical analysis post-retrieval of the implants

For immunohistochemical analysis of retrieved implants at day 3 and day 14 post-transplantation, sections of retrieved MNC were deparaffinized, hydrated and stained as described in earlier section. Antigen retrieval was performed using SDS. Non-specific binding was blocked using 1% BSA solution for 30 min at 37°C temperature and then incubated with primary antibodies such as β-tubulin III, MAP-2 specific to structural proteins and synapsin-1 for functional assessment of neuronal cells for overnight at 4°C. Following to the incubation, washing was done twice to remove unbound antibodies and further incubated with secondary antibodies for 60 min at room temperature. Immunofluorescence images were captured using inverted fluorescence microscopy (Car Zeiss, Germany).

### Immunophenotypic analysis of percentage neurological cells in MNC post-retrieval

The existence of percentage neuronal cells at day 3 and day 14 post-transplantation was quantified using immunophenotypic analysis of β-tubulin III protein expression in retrieved MNC. Briefly, cells on MNC were harvested using through trysinization process at day 3 and day 14 post-transplantation and stained with anti-human β-tubulin III antibody overnight at 4°C as described in earlier section. Following to staining, two step washing with 1X PBS was performed and cells expressing β-tubulin III protein were directly analyzed using Cell Quest software in Flow Cytometry.

### Statistical analysis

The results are represented as mean ± standard error of mean (SEM). The results of cytokine analysis are represented as fold values. The statistical significance was determined by one way and two way analysis of variance using ANOVA in Graph Pad Prism software (Version 5). Fold values of gene expression was calculated using 2^−ΔΔ*Ct*^ method. R programming was used for statistical computing and graphical representation of fold difference values of different mRNA transcripts analyzed by RT-qPCR. The data were compiled and run on a UNIX platform during R programming (R Version 3.1.2). The differences with *p*-value of < 0.05 was considered statistically significant.

## Ethics statement

This study was carried out in accordance with the recommendations of ICMR, India, Institutional Ethics Committee, Deccan College of Medical Sciences, Hyderabad with written informed consent from all subjects. All subjects gave written informed consent in accordance with the Declaration of ICMR criteria. The protocol was approved by the Institutional Ethics Committee of Deccan College of Medical Sciences, Hyderabad, Telangana, India.

## Author contributions

SV, SP, and AK conceptualized, designed the study and arranged the infrastructure and materials required to conduct the study. SV, AB, and CL performed experiments and wrote the manuscript. SV performed the statistical analysis and prepared the figures and designs. SV and CL formatted the manuscript.

### Conflict of interest statement

The authors declare that the research was conducted in the absence of any commercial or financial relationships that could be construed as a potential conflict of interest.
