# Supplementary material for: Bioengineering Human Neurological Constructs Using Decellularized Meningeal Scaffolds for Application in Spinal Cord Injury
Source: Front Bioeng Biotechnol. 2018 Nov 1;6:150. doi: 10.3389/fbioe.2018.00150 (PMC6221909; doi:10.3389/fbioe.2018.00150)
Supplement: Supplementary file 1 [file Data_Sheet_1.PDF]

SUPPLEMENTARY DATA

Bioengineering human neurological constructs using decellularized meningeal scaffolds for application in spinal cord injury

Sandeep Kumar Vishwakarma, Avinash Bardia, Chandrakala Lakkireddy, Syed Ameer Basha Paspala, Aleem Ahmed Khan*

Central Laboratory for Stem Cell Research and Translational Medicine, Centre for Liver Research and Diagnostics, Deccan College of Medical Sciences, Kanchanbagh, Hyderabad-500058, Telangana, India

Dr. Habeebullah Life Sciences, Attapur, Hyderabad, Telangana, India

***Corresponding Address**

Dr. Aleem Ahmed Khan
Central Laboratory for Stem Cell Research and Translational Medicine,
Centre for Liver Research and Diagnostics,
Deccan College of Medical Sciences,
Kanchanbagh, Hyderabad-500058,
Telangana, India
Ph/Fax: +91-40-24342954
Email: aleem_a_khan@rediffmail.com

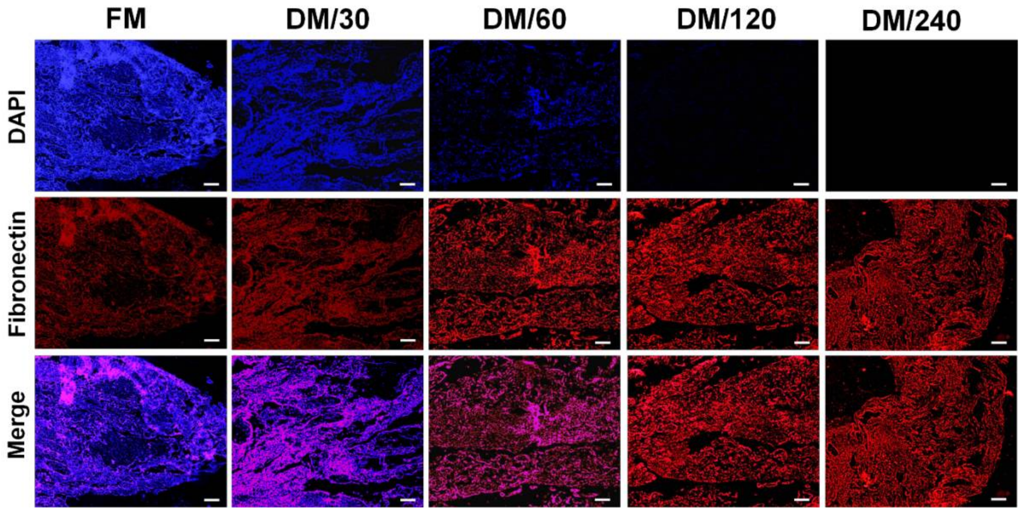

SFig. 2.1 Fibronectin staining of native (FM) and DMS showing intact ECM and gradual decrease in nuclear content (blue-DAPI staining) with increasing the time of decellularization. The nuclear staining was completely absent in decellularized meninges after 240 min (DM/240)

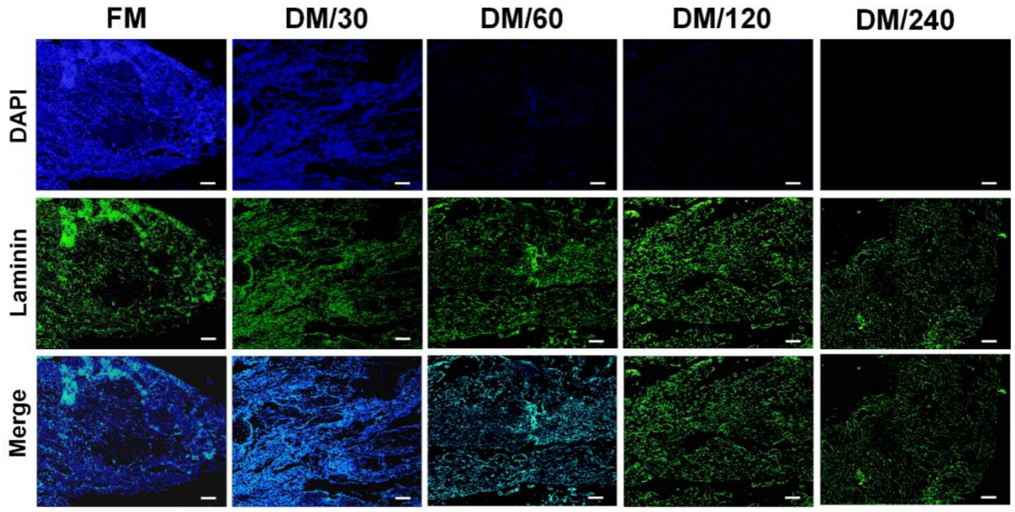

SFig. 2.2 Laminin staining of native (FM) and DMS showing intact ECM and gradual decrease in nuclear content (blue-DAPI staining) with increasing the time of decellularization. The nuclear staining was completely absent in decellularized meninges after 240min (DM/240)

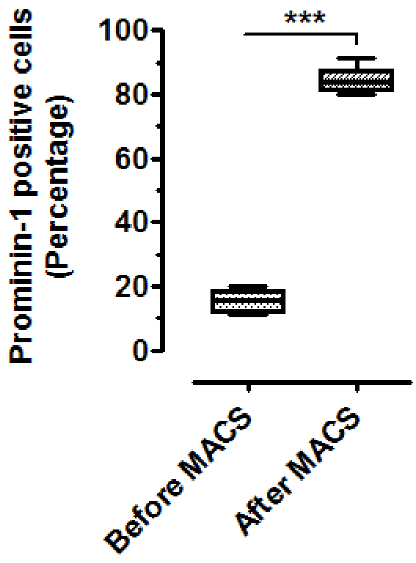

SFig. 4.1 Percentage prominin-1 positive cells before and after MACS

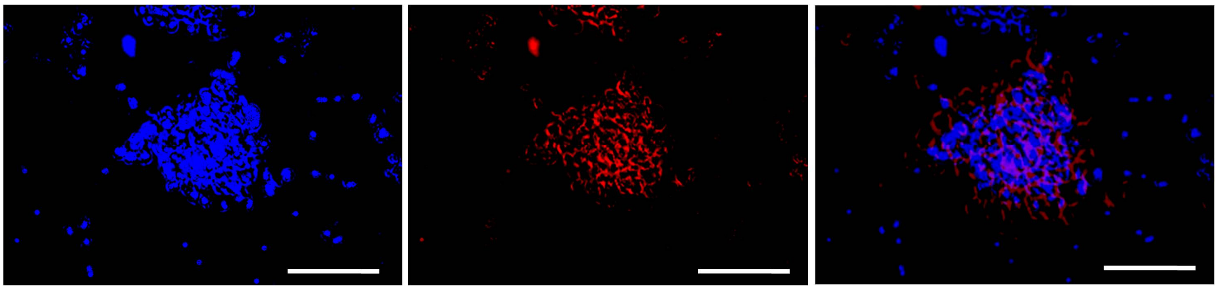

SFig. 4.2 Percentage hNPCs expressing Nestin within neurosphere derived from prominin-1-ve enriched cells

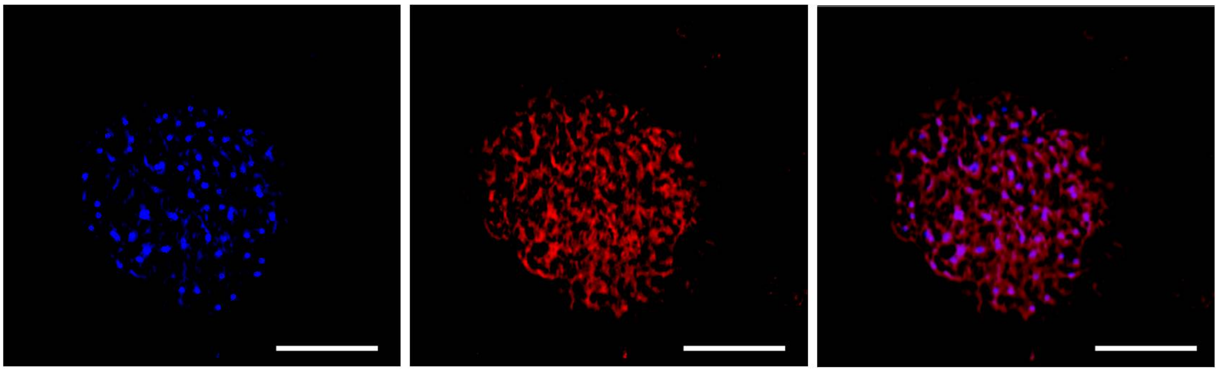

SFig. 4.3 Percentage hNPCs expressing Nestin within neurosphere derived from prominin-1+ve enriched cells

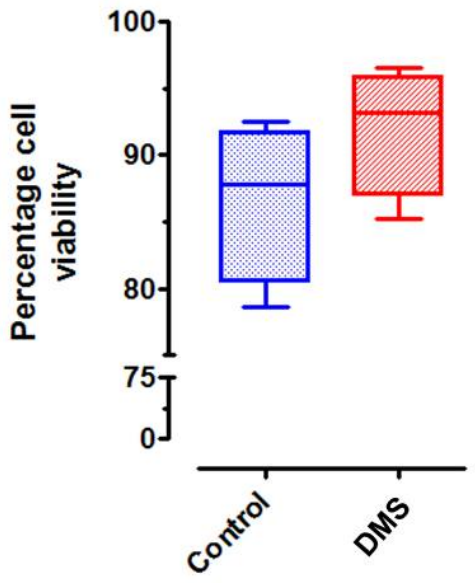

SFig. 4.4 The viability of neuronal cells cultured on DMS was higher than the control condition

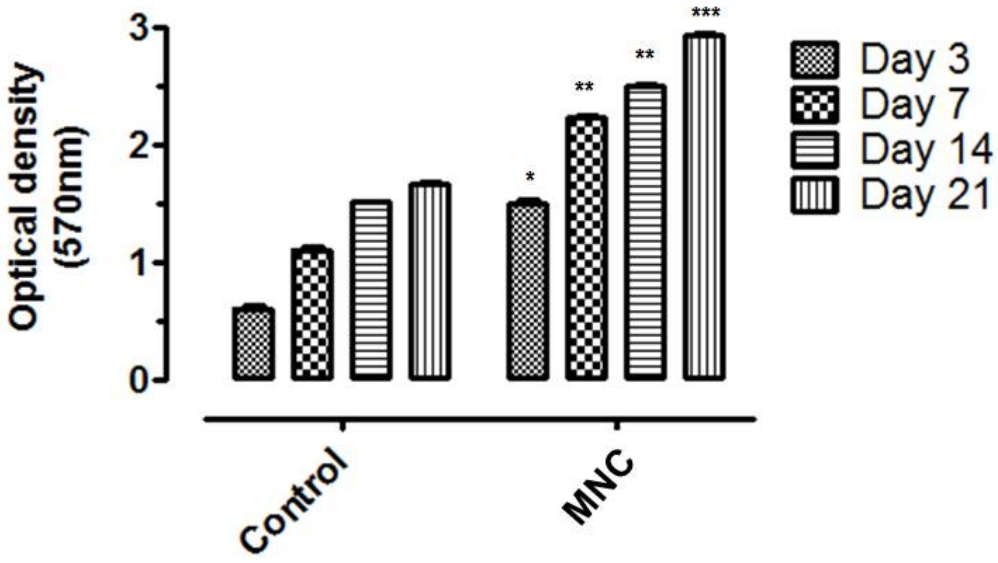

SFig. 4.5 MTT reduction assay showing higher degree of neuronal cells expansion on DMS as compared to the control

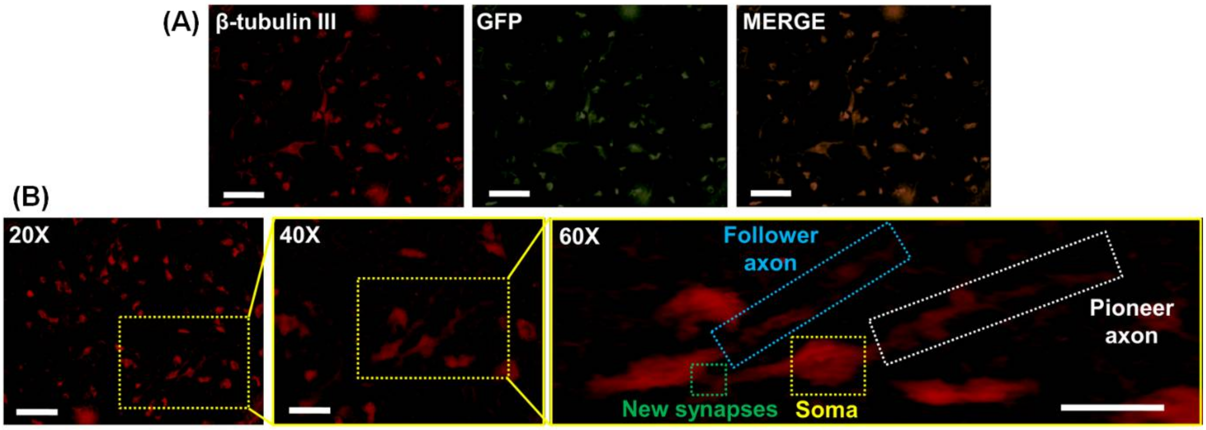

SFig. 4.6 Immunofluorescence staining of differentiated neurons on DMS showing **(A)** positive expression of neuronal specific marker β-tubulin III (**Red**) and green fluorescent protein (GFP, **Green**) (Scale bar: 40μm; Resolution: 10X) **(B)** Long axonal outgrowths along the pioneer axonal tract and formation of new synapses due to spatial configuration of neurons mimicking with the natural architecture and pattern (Scale bar: 50μm; Resolution: 100X)

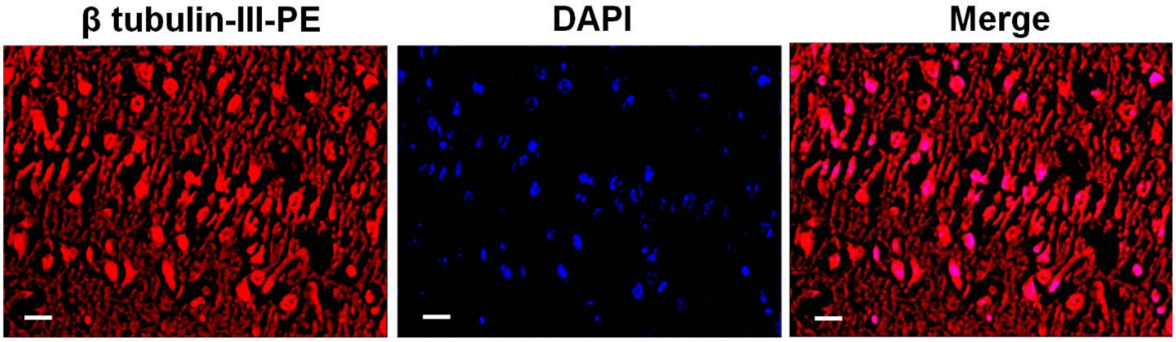

SFig. 4.7 β-tubulin III (red) and DAPI (blue) staining of neurons culture on DMS at day 14 showing well organized and aligned expansion of neuronal cells with new synapses, long axons and dendrites formation (Scale bar: 50μm; Resolution: 20X)

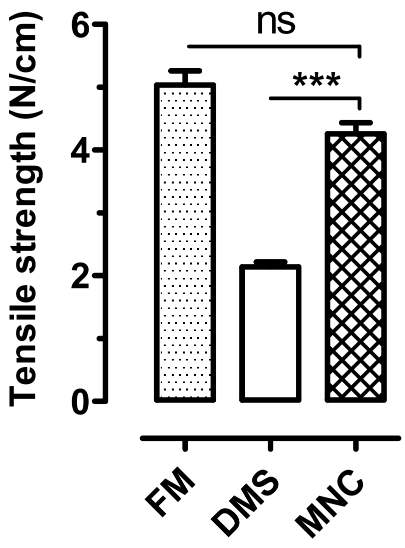

SFig. 4.8 Plot showing tensile strength of FM, DMS and MNC

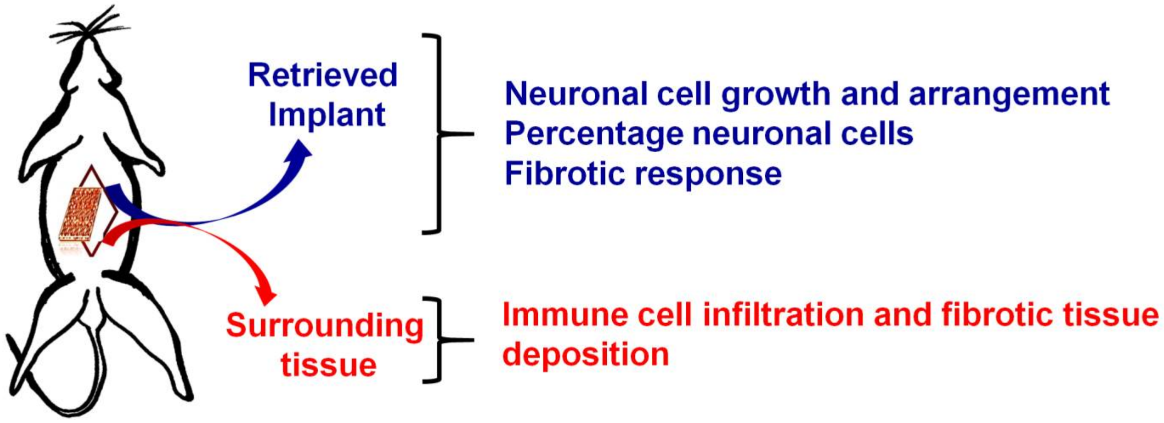

SFig. 6.1 Schematic representation showing biocompatibility studies of the DMS and MNC implants in rats to determine the immunological and fibrotic reactions

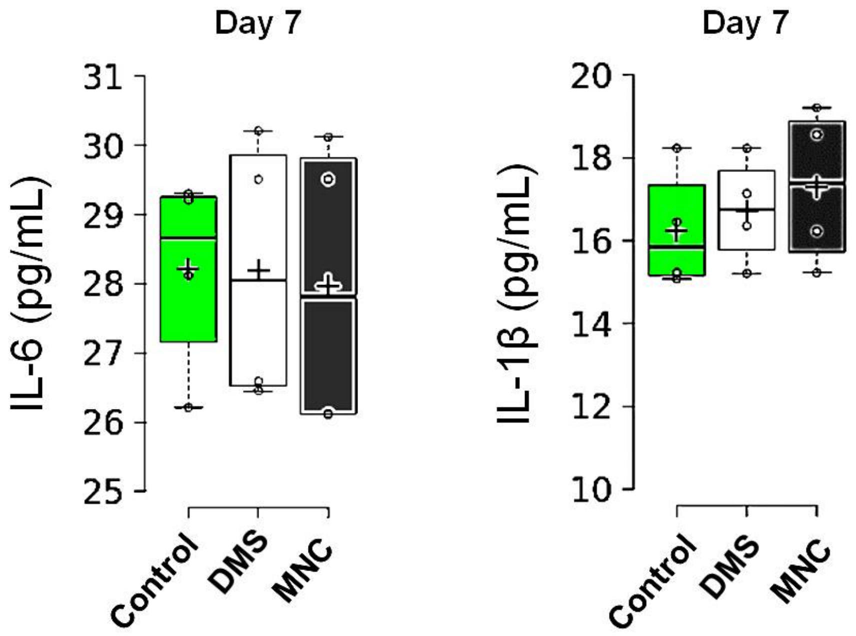

SFig. 6.2 The level of inflammatory cytokines IL-6 and IL-1β at day 7 post-transplantation of the DMS and MNC in rats

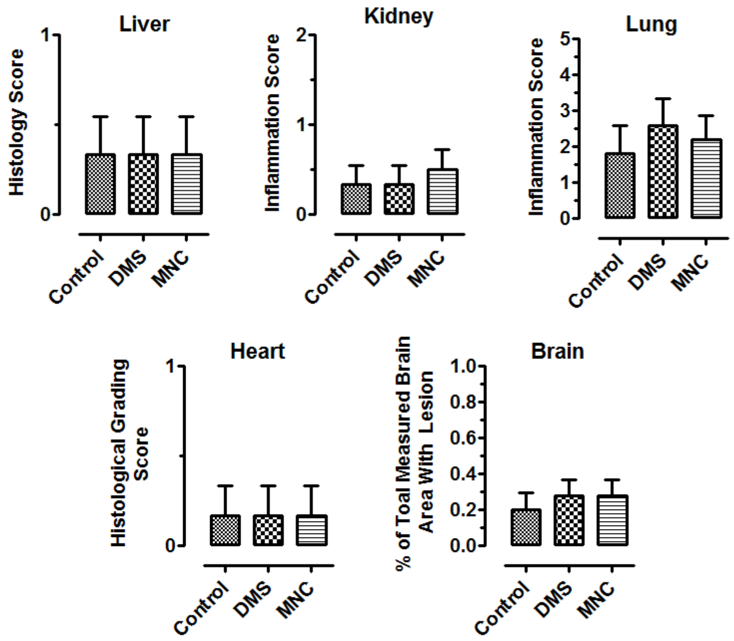

SFig. 6.3 Histological grades assigned to vital organs of rats post-transplantation of DMS and MNC at day 3

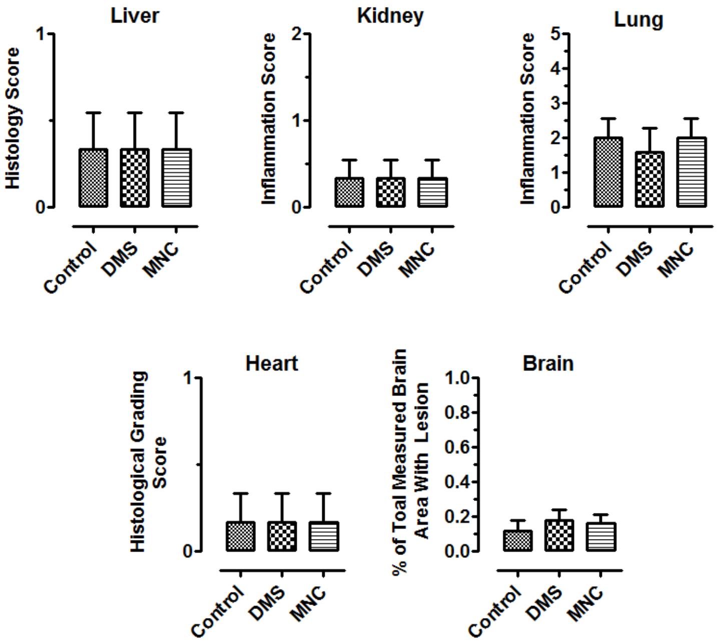

SFig. 6.4 Histological grades assigned to vital organs of rats post-transplantation of DMS and MNC at day 14

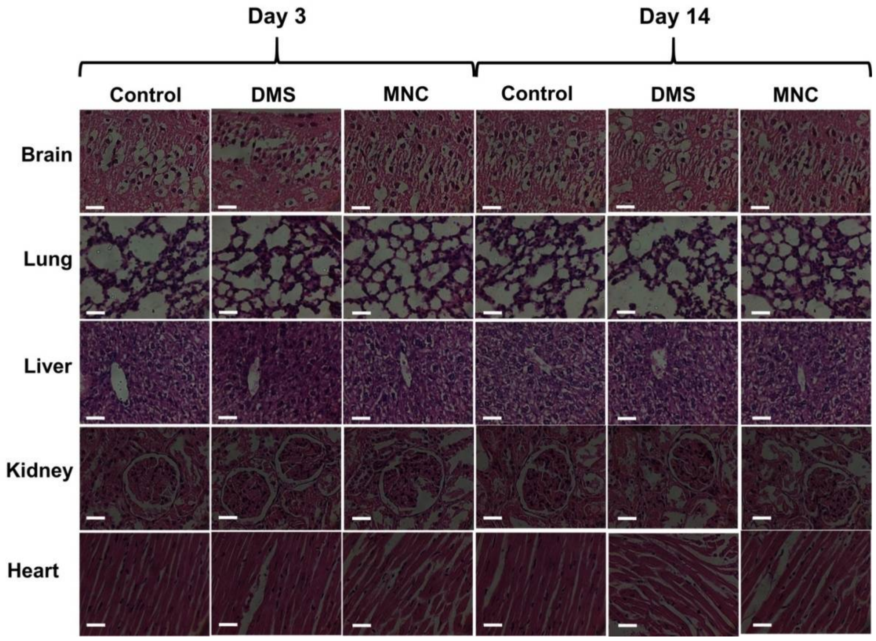

SFig. 6.5 Histopathological investigation of major vital organs (brain, lung, liver, kidney and heart) of rats at day 3 and 14 post-transplantation of DMS and MNC. The analysis revealed no significant change in histological features post-transplantation as compared to the control

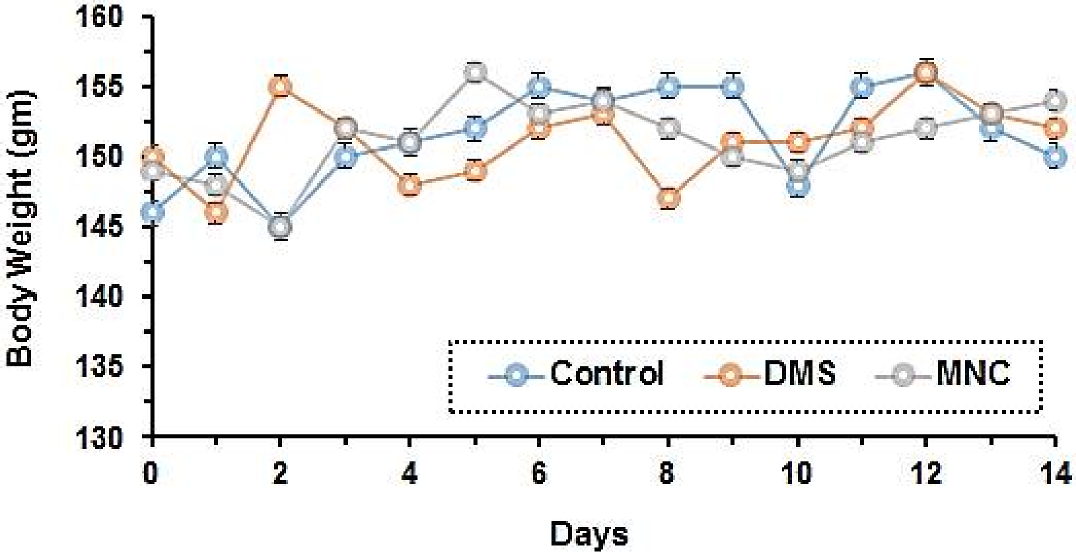

SFig. 6.6 No significant change was observed in animal body weight after transplantation of DMS as well as MNC as compared to control

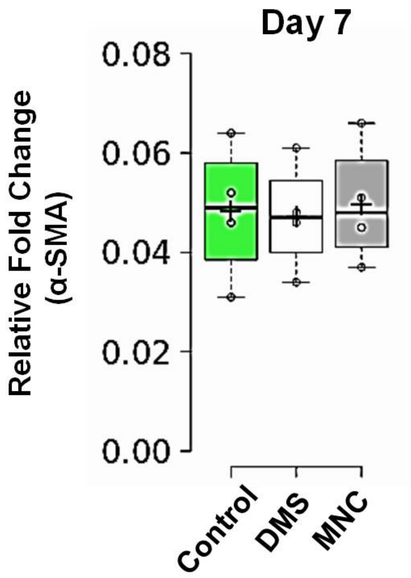

SFig. 7.1 Plot showing the change in expression level of α-SMA transcript in control and retrieved implants at post-transplantation day 7

STable 1 Changes in haematological parameters of rat at day 3 post-transplantation

	Control	DMS	MNC
**WBC (10^3/mm^3)**	10.5±1.049230485	10.51±1.705033748	10.15±0.471880215
**RBC (10^6/mm^3)**	8.34±0.564256258	8.32±1.05077749	8.255±0.533012702
**Neutrophils (10^3/mm^3)**	2.015±0.24671361	2.705±0.61384174	2.675±0.348260616
**Basophils (10^3/mm^3)**	0.021±0.021547005	0.022±0.011547005	0.023±0.021547005
**Eosinophils (10^3/mm^3)**	0.036±0.006773503	0.035±0.023094011	0.037±0.033094011
**Monocytes (10^3/mm^3)**	0.027±0.004773503	0.025±0.005773503	0.026±0.021547005
**Lymphocytes (10^3/mm^3)**	6.8±0.688897275	7.14±1.254700538	6.39±0.163923048
**Hemoglobin (g/dL)**	15.5±0.446410162	15.45±0.504145188	13.95±1.312435565
**HCT (%)**	43.18±1.072324495	42.345±2.934789822	45.03±4.6033321
**Platelets (10^3/mm^3)**	238±68.13733176	272±108.9230485	370.5±36.21836642

STable 2 Changes in haematological parameters of rat at day 7 post-transplantation

	Control	DMS	MNC
**WBC (10^3/mm^3)**	10.42±0.219393102	10.56±1.270170592	10.19±0.461880215
**RBC (10^6/mm^3)**	7.52±0.011547005	7.45±0.138564065	7.92±1.235529576
**Neutrophils (10^3/mm^3)**	2.91±0.635085296	2.55±0.808290377	3.28±0.069282032
**Basophils (10^3/mm^3)**	0.025±0.017320508	0.025±0.005773503	0.022±0.011547005
**Eosinophils (10^3/mm^3)**	0.085±0.005773503	0.075±0.017320508	0.055±0.005773503
**Monocytes (10^3/mm^3)**	0.022±0.011547005	0.025±0.005773503	0.026±0.011547005
**Lymphocytes (10^3/mm^3)**	5.77±0.831384388	6.165±0.744781847	6.122±1.258623587
**Hemoglobin (g/dL)**	15.25±0.173205081	12.28±1.293264603	14.96±0.034641016
**HCT (%)**	46.65±6.17764788	55.13±14.74552588	55.125±2.580755703
**Platelets (10^3/mm^3)**	312.5±102.1909976	353.5±44.45597073	366±142.0281662

STable 3 Changes in haematological parameters of rat at day 14 post-transplantation

	**Control**	**DMS**	**MNC**
**WBC (10^3/mm^3)**	12.51±0.565685425	10.69±0.692964646	10.71±0.565685425
**RBC (10^6/mm^3)**	8.55±1.173797257	8.54±0.67882251	7.375±0.190918831
**Neutrophils (10^3/mm^3)**	2.515±0.714177849	1.98±0.183847763	2.14±0.028284271
**Basophils (10^3/mm^3)**	0.022±0.014142136	0.025±0.007071068	0.029±0.00421
**Eosinophils (10^3/mm^3)**	0.045±0.007071068	0.051±0.028284271	0.055±0.021213203
**Monocytes (10^3/mm^3)**	0.022±0.014142136	0.025±0.007071068	0.025±0.007071068
**Lymphocytes (10^3/mm^3)**	5.765±0.643467171	6.05±1.187939392	5.81±0.820243866
**Hemoglobin (g/dL)**	13.92±2.545584412	15.005±0.28991378	14.49±1.286934342
**HCT (%)**	38.45±0.926309883	38.6±0.848528137	51.75±0.777817459
**Platelets (10^3/mm^3)**	256±53.74011537	369.5±47.37615434	173.5±7.778174593

STable 4 Changes in liver function parameters of rat at day 3, 7 and 14 post-transplantation

**Albumin**	**Control**	**DMS**	**MNC**
**Day 3**	3.062± 0.2485	3.164±0.1567	3.184±0.1505
**Day 7**	3.254±0.2558	3.021±0.2226	2.810±0.1857
**Day 14**	2.750±0.2240	3.136±0.2651	3.319±0.3886
**ALP**			
**Day 3**	96.85±11.69	103.6±6.374	97.67±10.17
**Day 7**	97.81±9.763	84.27±9.853	95.67±12.36
**Day 14**	105.1±7.127	96.95±9.01	112.0±8.175
**Bilirubin**			
**Day 3**	0.2737±0.03765	0.2571±0.02923	0.2530±0.02636
**Day 7**	0.2572±0.02282	0.2796±0.02717	0.2649±0.03087
**Day 14**	0.2883±0.02468	0.2889±0.03933	0.2735±0.02795
**SGOT**			
**Day 3**	48.47±3.931	65.77±7.395	48.60±5.187

**Day 7**	55.48±7.405	55.05±7.862	56.95±7.068
**Day 14**	53.02±6.147	65.76±1.511	44.95±3.103
**SGPT**			
**Day 3**	25.57±2.726	24.57±2.076	23.68±2.450
**Day 7**	21.83±1.408	21.70±1.407	22.99±2.814
**Day 14**	20.37±2.697	22.81±2.284	19.77±4.367

STable 5 Changes in renal function parameters of rat at day 3, 7 and 14 post-transplantation

**Urea**	**Control**	**DMS**	**MNC**
**Day 3**	18.45±2.253	17.99±1.578	16.30±1.387
**Day 7**	18.59±1.034	18.14±1.607	18.35±1.261
**Day 14**	17.04±0.8221	18.52±0.9826	16.14±2.006
**Creatinine**			
**Day 3**	0.5446±0.02481	0.5880±0.03800	0.4083±0.02356
**Day 7**	0.6203±0.06122	0.6387±0.04176	0.5241±0.08814
**Day 14**	0.6479±0.05464	0.6331±0.02560	0.5629±0.07161
